# Impact of Interest Congruence on Study Outcomes

**DOI:** 10.3389/fpsyg.2022.816620

**Published:** 2022-03-04

**Authors:** Bernhard Ertl, Florian G. Hartmann, Anja Wunderlich

**Affiliations:** ^1^Learning and Teaching With Media, Department of Human Sciences, Universität der Bundeswehr München, Neubiberg, Germany; ^2^Methodology in the Social Sciences, Department of Human Sciences, Universität der Bundeswehr München, Neubiberg, Germany

**Keywords:** vocational interests, Holland model, university freshmen, congruence, vocational aspiration

## Abstract

Grounding on Holland’s RIASEC model of vocational interests and the respective assumptions on person-environment fit (congruence), this paper focuses on how congruence is related to study outcomes, especially students’ persistence, performance, and satisfaction. The paper distinguishes the measure of congruence with respect to social congruence (SOC) (interest fit with the study mates) and aspirational congruence (ASP) (interest fit with the occupation aspired) and also distinguishes the effects of congruence for gender and six different study areas including Science, Technology, Engineering, Mathematics (STEM), medicine, economics, education, and languages. The paper analyses 10,226 university freshmen of the German National Educational Panel Study (NEPS) and follows them longitudinally with respect to their study outcomes. The results show that students’ persistence was more related to SOC than to ASP, especially for male students. Furthermore, SOC was particularly important for students in STEM areas. Regarding performance, however, ASP was more important. Here, we notably found correlations for STEM subjects with a balanced proportion of female students. Regarding satisfaction, mainly marginal correlations could be found. The results indicate conceptual differences between social and aspirational congruence as well as specific effects for gender and study area. While research might take this into account by specifically developing their models for different study areas, career counseling may reflect on the different significance of the interest-based person-environment fit for different study areas. Initiatives for raising young people’s participation in STEM should therefore specifically focus on students that have high chances to develop interest profiles that are congruent to STEM rather than students who show profiles which already indicate a low congruence.

## Introduction

Choosing an academic major and a corresponding occupation are important decisions determining students’ further course of life (Elder, 2002). Person-environment fit (P-E fit) theories suggest that career-related choices are promising if they are based on individual traits (Su et al., 2015) such as vocational interests as defined by Holland (1997). Being one of the most prominent P-E fit approaches (Ott-Holland et al., 2013; Juntunen et al., 2019), Holland’s (1997) RIASEC model claims that students who choose an environment that is congruent to their interests should be satisfied, perform well, and persist. Incongruent choices, on the other hand, are supposed to ultimately lead to students dropping out, which causes personal costs such as a lower self-esteem (Hoeschler and Backes-Gellner, 2017) or forgone earnings (Schneider and Yin, 2011) as well as societal costs such as resources invested in vain at institutions of higher education, a lack of skilled professionals, or the loss of tax revenues (Sarcletti and Müller, 2011; Schneider and Yin, 2011; Neugebauer et al., 2019). On an individual level, dropping out can be a sensible decision at some point in time and may result in a degree in another (and better fitting) subject area (Donohue, 2006), but picking an optimal environment right from the start appears to be the better option (Allen and Robbins, 2008). Hence, research looks for preventive measures to avoid dropouts or detours, and interest congruence is considered a decisive predictor.

Nye et al. (2012) carried out a meta-analysis and found a moderate effect of interest congruence on grades and persistence in academic samples. Results of meta-analyses and reviews investigating the effect on satisfaction show rather small correlations especially in academic settings (Assouline and Meir, 1987; Tranberg et al., 1993; Tsabari et al., 2005; Hoff et al., 2020). Recent studies tried to explain such weak relations by moderators, most of which can be assigned to one of three variable types: personal characteristics such as age or gender, characteristics of the environment (such as homogeneity of the work environment regarding values or interests), and methods to conceptualize or measure interest congruence (Fu et al., 2019).

### Congruence in Stereotyped Domains

While such studies provide evidence for the effects of congruence in general, much less is known about the effects of congruence for females and males in stereotyped domains like STEM (Science, Technology, Engineering, Mathematics). Gottfredson (2005) introduced the *sextype* of an occupation that may be male, neutral, or female and discussed that individuals may rule out some occupations because of having the wrong sextype. She calls it as a high level of concern if students compromise their interests (respectively their interest congruence) for going into a profession with a better fitting sextype. This may especially apply for the STEM area that has several subjects showing an under-representation of female students (see e.g., Ertl et al., 2017), but also in the education area for male students. Regarding STEM, we focus on the narrow definition also applied in Ertl et al. (2017) that primarily includes the natural sciences, because these are usually considered of having a male sextype. This is different for medicine that is recently more and more seen as a female domain. However, STEM can further be distinguished, analogous to Ertl et al. (2017), in subjects with a low proportion of female students (STEM-L with below 30% of female students, e.g., physics, engineering) for which the sextype *male* corresponds to the proportion of female students and subjects with a medium proportion of students (STEM-M with 30–70% female students) with subjects like mathematics that still are perceived as male although the proportion of male and female students is almost balanced. Distinguishing these areas has some implications e.g., for measures and interventions for promoting females going into STEM. Interest based occupational inventories for career development, e.g., the EXPLORIX (Jörin Fux et al., 2003) or the SDS (Holland et al., 1973), allow to identify female students with good chances for persisting STEM and help thereby to focus measures and interventions on these students that respond best on them. While for students in STEM-M, the regular set of measures may be sufficient because they find plenty of same-sex mates in the courses, this is different for female students in STEM-L that often feel belonging uncertainty (see e.g., Deiglmayr et al., 2019; Höhne and Zander, 2019). Thus, measures for STEM-L may therefore need an additional focus on mentoring (e.g., Kricorian et al., 2020) and networking.

The current study contributes to the investigation of the congruence-outcome relation in academic settings to evaluate how far this measure can inform interventions to select adequate candidates. As academic majors differ with regard to multiple characteristics such as career choice motivations, career choice options, or barriers that have to be overcome (e.g., gender stereotypes), the impact of congruence is supposed to be different for different majors (Tracey et al., 2012; Le et al., 2014; Etzel and Nagy, 2016; Nguyen et al., 2016; Schelfhout et al., 2019b). Therefore, the paper aims at disclosing the effect of interest congruence on study outcomes in different majors including STEM fields with different gender distributions. Building upon recent studies, a holistic investigation is carried out, which includes applying different conceptualizations of congruence and using a sophisticated method to measure congruence and longitudinal large-scale data comprising 14 waves and 8 years.

## Holland’s Person-Environment Fit Theory

The RIASEC model (Holland, 1997) describes vocational interests using six different dimensions comprising preferences for specific activities. The *Realistic (R)* dimension includes the preference for practical and technical activities producing concrete results, the *Investigative (I)* dimension comprises of the interest in intellectual and scientific activities studying complex problems, the *Artistic (A)* dimension includes favoring open and unstructured activities demanding creativity and yielding art products or forms, the *Social (S)* dimension consists of social activities like teaching or caring, the *Enterprising (E)* dimension contains the preference for activities that aim at convincing or manipulating other people by verbal or other means, and the *Conventional (C)* dimension conclusively comprises of the interest in regular activities such as the orderly and systematic handling of data. These six dimensions are usually called RIASEC dimensions and are not only used to describe people but also work-related environments such as occupations or majors so that both people and environments can be assigned a RIASEC interest profile. According to Holland’s (1997) *calculus hypothesis*, the six dimensions are not independent from each other but can be arranged hexagonally with their spatial distances indicating their psychological similarity. For example, the Social type is more similar to the Enterprising type than to the Conventional type and it is antagonistic to the Realistic type. There is broad evidence for the calculus hypothesis in U.S. samples (Tracey and Rounds, 1993; Rounds and Tracey, 1996) as well as for other countries such as Germany (Nagy et al., 2010).

The *congruence hypothesis* now states that people who choose an environment of the RIASEC profile which is similar to their personal RIASEC profile show favorable outcomes like satisfaction, performance and persistence. This hypothesis has been comprehensively studied and, overall, has been well confirmed by meta-analyses (e.g., Van Iddekinge et al., 2011; Nye et al., 2012, 2017; Hoff et al., 2020). As mentioned before, there are indications that the congruence-outcome relation is influenced by several moderators. For example, there are manifold methods to conceptualize and to measure congruence affecting the magnitude of the congruence-outcome correlation (e.g., Assouline and Meir, 1987).

According to Muchinsky and Monahan (1987) two *concepts of congruence* can be distinguished. The first, *supplementary fit*, is the similarity between the individual and the people in the environment. The second, *complementary fit*, focuses on the resources or demands of an environment and is given if a person’s abilities meet these demands or if a person’s needs are met by the supplied resources (Kristof-Brown et al., 2005; Su et al., 2015). Scholars disagree on whether interest congruence is more of supplementary or complementary nature. For example, while Henry (1989) states that “Holland’s theory is the prototypical supplementary model in which individuals pursue careers which supplement their interests” (p. 38), there are indications that interest congruence follows a rather complementary fit approach (Wiegand et al., 2021).

Regarding the *measurement of fit*, there is a distinction of *direct and indirect measures* (Kristof-Brown et al., 2005). A direct measure would be the perceived fit of an individual. Indirect measures require the assessment of both the person’s and the environment’s characteristics. In addition, indirect measures can be *subjective*, when the characterization of both the person and the environment are based on self-reports by the same person, or *objective*, when the description of the person and the environment are based on different sources. With respect to objective measures, the assessment of the environment can be based on the aggregated interests of the people in the environment (*incumbents*) or on the judgments by *experts* (Rounds et al., 1999). Once the data of the person and of the environment are available, no matter whether the subjective or objective approach was followed, congruence in the context of Holland’s (1997) theory can be calculated alternatively with more recent measures being superior. Here, older *measures* of congruence use only three or less RIASEC dimensions that are dominant for a person or an environment, respectively (Camp and Chartrand, 1992; Brown and Gore, 1994). These measurement approaches have been criticized since the full RIASEC interest profile information is not used and potential outcome-relevant information is ignored (Dik et al., 2010; Tracey and Sodano, 2013). Measures that are recently used take all six RIASEC dimensions into account with the *Euclidean distance* being especially theoretically grounded since it also counts in the aforementioned calculus hypothesis. Following the assumption that a RIASEC profile can be represented by a vector in a two-dimensional space (Prediger, 1982; Eder, 1998), the Euclidean distance measures the distance between the personal RIASEC vector and the environmental RIASEC vector so that smaller values indicate higher congruence. In the current study, the Euclidean distance is used to measure congruence.

## Meta-Analytic Findings on the Effect of Interest Congruence on Academic Outcomes

Satisfaction, performance, and persistence are central education- and work-related outcomes whose relation to vocational interests has been widely studied. Some of the reviews and meta-analysis summarizing this research focused solely on the occupational context (Van Iddekinge et al., 2011; Hoff et al., 2020) while others also carried out analyses for academic environments. This has been done with regard to the association of interest congruence with satisfaction (Assouline and Meir, 1987; Tranberg et al., 1993; Tsabari et al., 2005) as well as with performance and persistence (Nye et al., 2012).

### Performance and Persistence

Considering academic samples, Nye et al. (2012) summarize over 60 years of research on the effects of vocational interests on performance and persistence. Overall, they found that interests correlated moderately with performance and persistence for employed samples as well as for academic samples (baseline corrected correlations of interest congruence with performance and persistence of 0.30 and 0.36 in employed samples and 0.30 and 0.34 in academic samples). For the current investigation, we took a closer look at those studies that analyzed interest congruence in academic samples and were explicitly based on Holland’s (1997) theory. Here, we focused on three variables that could moderate the congruence-outcome relation, namely methods to measure congruence, gender (as a personal characteristic), and diversity of majors (as a characteristic of the environment) (see Fu et al., 2019).

With regard to the measurement of congruence, the studies summarized by Nye et al. (2012) predominantly used dated methods. For example, Chartrand et al. (1992) used the M Index (Iachan, 1984), which considers only the three dominant RIASEC dimensions of the person and the three dominant dimensions of the environment. Other studies even used only one dimension of the person and one dimension of the environment and ignored the remaining RIASEC dimensions (Bruch and Krieshok, 1981; Henry, 1989). Since such studies do not use full interest profile information to depict the interest structure of the person and the environment, they may be a suboptimal database to estimate the effects of interest congruence. They are furthermore able to provide only ordinal congruence estimations (see Hartmann et al., 2021). As a consequence, the congruence-outcome relation could be different when sophisticated methods are used. Based on the meta-analysis by Nye et al. (2012), this question remains unanswered.

With respect to gender, some studies investigated only males (Bruch and Krieshok, 1981) or only females (Camp and Chartrand, 1992). Studies examining both male and female students mostly found similar effects (Henry, 1989; Leuwerke et al., 2004). However, the study by Nichols and Holland (1963) found interest-outcome relations varying with gender. For example, the relation between the preference for realistic occupations and literary achievement was –0.37 for males and –0.08 for females while the relation between the preference for realistic occupations and (rare) scientific achievement was –0.06 for males and 0.43 for females.

Focusing on the diversity of majors in the samples, part of the studies investigated students from only one single academic major and came to different conclusions about the congruence-outcome relation. For example, Chartrand et al. (1992) only analyzed the vocational interests of psychology students and found no relation between interest congruence and students’ GPA in psychology. Bruch and Krieshok (1981) studied students with engineering majors especially requiring Investigative interests. They only considered students who showed high Realistic or high Investigative interests. In line with the congruence hypothesis, students with high Investigative interests showed higher persistence and also attained better grades than students showing high Realistic interests. These studies focusing on different, and in each case, homogenous groups of students indicate that the academic major might be a moderator of the congruence-outcome relation. Studies considering multiple academic majors (e.g., Schmitt et al., 2008) yielded significantly lower effects compared to studies focusing a single major (Nye et al., 2012).

Overall, the meta-analysis by Nye et al. (2012) indicates that interest congruence is an important predictor of performance and persistence in the academic context and can be a preventive factor with regard to dropouts or detours. However, many studies summarized, used dated and rather coarse methods to measure congruence, and it remains unclear whether the effects are equal for male and female students in different academic majors.

### Satisfaction

With regard to the relation between interest congruence and satisfaction, a few reviews and meta-analysis have been carried out (Assouline and Meir, 1987; Tranberg et al., 1993; Tsabari et al., 2005). These investigations found no significant correlations within the occupational context and negligible or non-significant correlations between interest congruence and satisfaction in the academic context. In detail, Assouline and Meir (1987) found a mean correlation of 0.098 based on six correlations, Tranberg et al. (1993) found a mean correlation of 0.095 based on five correlations, and Tsabari et al. (2005) only cited one study analyzing academic satisfaction, which showed a correlation of –0.033. Recently, Hoff et al. (2020) criticized these meta-analyses and carried out another meta-study overcoming some weaknesses of the criticized ones. For example, they analyzed far more studies resulting in sufficient statistical power and revealing a statistically significant correlation between interest fit and job satisfaction of 0.19 (95% CI: 0.16,0.21). Unfortunately, they did not consider studies investigating academic samples.

To sum up, according to previous meta-analyses, evidence for the congruence-satisfaction relation is weak; however, this might be due to methodological issues or other moderating variables such as gender or major. The results regarding performance, persistence, and satisfaction also indicate that interest congruence may have effects of different strengths on different academic outcomes, namely a stronger effect on performance and persistence than on satisfaction.

## Recent Evidence

With few exceptions, current studies use sophisticated methods to measure congruence such as profile correlation or the Euclidean distance, which take all six RIASEC dimensions into account (e.g., Tracey et al., 2012; Nguyen et al., 2016; Kim and Beier, 2020). These studies found interest congruence to be related to performance (Tracey et al., 2012; Nye et al., 2018), major persistence (Allen and Robbins, 2008; Tracey et al., 2012; Le et al., 2014; Le and Robbins, 2016; Nguyen et al., 2016; Kim and Beier, 2020), and major satisfaction (Bai and Liao, 2019). Studies that took gender as a moderator into account indicated that the effects of interest congruence on persistence (in STEM) is similar for men and women (Le et al., 2014; Le and Robbins, 2016). As with studies considering gender as a moderator, studies considering academic majors as a moderator are rare. Tracey et al. (2012) focused only on a specific aspect of academic majors, namely environmental constraint (i.e., interest homogeneity within an academic major), and concluded that the relation between interest congruence and academic outcomes is stronger for majors that are more constraint, i.e., show more homogeneity regarding the RIASEC profiles of the people in the majors (incumbents). Le et al. (2014) carried out separate analyses for two groups of STEM majors: STEM Science and STEM Quantitative (technology, engineering, and mathematics). Although they found similar main effects of interest congruence on persistence, effects regarding the interaction between abilities and interest fit were different. Nguyen et al. (2016) carried out separate analyses for students of biology and students of chemistry. Using the Euclidean distance as a congruence measure, they only found interest congruence to be related to persistence for chemistry but not for biology. Based on their results Nguyen et al. (2016) concluded that “the factors that impact university success and retention vary across majors, complicating attempts to address retention efforts at universities; a broad, discipline-unspecific approach will not suffice” (p. 12). In addition, it is possible that two moderating variables, such as gender and major, interacted with one another. For example, Le et al. (2014) referred to Heilman et al. (2004) and argued that women in STEM fields have to overcome barriers such as gender stereotypes including negative reactions in case of success regarding tasks that are male gender-typed. These preconditions (or external factors) could weaken the role of interest congruence and its impact on academic outcomes for female students in such majors (see also Lent, 2013; Fu et al., 2019).

## The Current Study

To sum up, recent research gives some indications that the effects of congruence on academic outcomes vary with respect to gender and subject area although systematic comparisons are missing so far. To shed light on this area, this study will analyze the effects of congruence with a specific focus on gender and subject area. Our main focus lies on the STEM areas with a low and medium proportion of female students, but we will contextualize these results with further areas for being able to discuss whether the effects revealed are STEM specific or rather general. Therefore, the present study grounds its analyses on a national large-scale study that enables the analysis of effects in general as well as specifically with respect to gender and different academic areas. The present study includes two conceptualizations of congruence: social congruence (SOC) that depicts the congruence between the interests of the individual and the interests of the respective mates in the study subject (incumbents) and aspirational congruence (ASP) that depicts the congruence between the individual’s interests and the professions aspired. For both conceptualizations, a sophisticated method is used to quantify the extent of congruence by calculating the Euclidean distance, which is grounded on Holland’s (1997) RIASEC model and considers full profile information. This also allows a better estimate of mean differences, e.g., between persisters and non-persisters rather than only rank-order correlations. The study furthermore covers several outcome measures like completing the studies, the respective grades, and study satisfaction.

## Research Questions

This study analyses the effect of interest congruence (at study entry) on study outcomes. The approach thereby is to focus first on the whole cohort, then specifically on male and female students, and then on differences between the study areas.

The first research question focuses students’ *persistence*, i.e., if students finished their degree program successfully, if they failed, or if they left their studies with a different state. As these states of persistence are categorical variables, RQ 1 analyzes to what extent students of these persistence state groups distinguish with respect to their congruence. The respective research question is:

RQ1: To what extent do students with different states of persistence distinguish with respect to social and aspirational congruence?

RQ1a: To what extent do male and female students with different states of persistence distinguish with respect to social and aspirational congruence?
RQ1b: To what extent do students of different subject areas with different states of persistence distinguish with respect to social and aspirational congruence?

*Hypothesis 1*: Previous research, summarized by the meta-study of Nye et al. (2012) provided evidence for correlations between congruence and persistence. Therefore, we hypothesize successful/persisting students being more congruent than less successful. Although literature indicates effects for gender (Le et al., 2014) and study subject (Nguyen et al., 2016), the body of previous research is not strong enough to deduct clear hypotheses regarding either of both aspects.

Besides persistence, also performance will be considered:

RQ2: To what extent do social and aspirational congruence correlate with study grade (*performanc*e)?

RQ2a: To what extent do social and aspirational congruence correlate with study grade for male and female students?
RQ2b: To what extent do social and aspirational congruence correlate with study grade for the different study areas?

*Hypothesis 2*: According to Nye et al. (2012), we expect that congruence correlates positively with study grade (performance).

The third research question will focus on satisfaction:

RQ3: To what extent is students’ satisfaction related to social and aspirational congruence?

We will investigate this research question for male and female students (RQ3a) as well as for students from different subject areas (RQ3b). With respect to satisfaction, we would expect positive correlations in accordance with Holland’s (1997) congruence hypothesis which means that a better congruence is correlated with a higher satisfaction. However, previous research showed ambiguous results in this regard. Therefore, we avoided stating a clear hypothesis and instead assumed that study subjects and gender may be a key to interpret the ambivalent results.

## Materials and Methods

The data set for this study comes from the German National Educational Panel Study (NEPS; Blossfeld et al., 2011; see also acknowledgments) and its starting cohort 5 that focuses on first year students (SC5:14:0.0). This cohort started their studies in autumn 2010 (FDZ-LIfBi, 2021b), and is still actively being followed. Students entered the survey with wave 1 in autumn 2010 and were then surveyed in each wave either per computer assisted telephone interview (CATI), computer assisted web interview (CAWI), or, e.g., for competence tests, sometimes a part of the students was tested in presence. An overview on the survey waves and times of survey can be found at FDZ-LIfBi (2021b). Some student information does not fit into the wave format and is represented in an episode format in the dataset, e.g., participation in study programs, internships, or work contracts. These were coded as episodes with a starting point and an end date, the study program in which a student participated and a status of termination of a study program (see FDZ-LIfBi, 2021a,b). Thus, if a student was enrolled in two study programs concurrently then the episode data would contain two episodes for these students with overlapping dates. If students mentioned that they dropped out, they were given a specific reason for dropout questionnaire^1^. However, the data set contains a notable number of students with open episodes; for them it remains unclear if they dropped out, if they finished their studies, or if they were unavailable for other reasons. We will tackle this phenomenon by focusing on two different measures for persistence: (a) we first will analyze whether students finished their initial study episode successfully or whether they failed and (b) then we will check how far students finished any study episode successfully or whether they mentioned that they dropped out.

The current data set includes 14 survey waves, and wave 14 was surveyed in autumn 2018. The study analyzed several variables that were surveyed in specific waves, especially interests (wave 1), aspirations (wave 1), and satisfaction (wave 3 that was collected in spring, 2012 after about 18 months of study, and wave 5 that was collected one year later in spring 2013; FDZ-LIfBi, 2021b). The dataset also included event specific variables like the completing or failing of a study episode with its respective final grade. This longitudinal perspective was essential for being able to reveal long time effects but suffers from issues like panel attrition, which resulted in far less students providing information in wave 14.

### Sample

Within this NEPS cohort of first year students, the study specifically focuses on students with the birth years from 1988 to 1991 to have a homogeneous age range that includes the majority of first year students (79%). Consequently, they were aged between 18 and 22 at study entry and respectively between 21 and 25 at wave 5. Within this age range, the study further narrows its focus on six major study areas: STEM with a low proportion of female students (STEM-L; less than 30% female students; 2,979 students; e.g., physics, engineering, computer science; see Supplementary Material 1), STEM with a balanced proportion of male and female students (STEM-M; between 30 and 70% female students; 2,457 students; e.g., mathematics, biology, chemistry), medicine (498 students; balanced; mainly general medicine and dentistry), economics (1,369 students; balanced; only the group of economics), education with a high proportion of female students (723 students; more than 70% female students; including education and welfare), and languages (2,200 students; high; German, English, and Roman language). In total, the study includes 10,226 students, 4,269 males and 5,957 females which are 57% of the initial sample.

### Congruence Measures

Both congruence measures build on an assessment of vocational interests. These were surveyed at wave 1 by the Interest Inventory Life Span (IILS-II scales; von Maurice and Nagy, 2009; Wohlkinger et al., 2011; see also FDZ-LIfBi, 2021a, pp. 699–704). This inventory covered each dimension with three items in a Likert scale format. Internal consistencies varied from α = 0.523 to α = 0.749 (Cronbach’s α for Realistic: α = 0.704; Investigative: α = 0.625; Artistic: α = 0.629; Social: α = 0.749; Enterprising: α = 0.523; Conventional: α = 0.561). Considering that these α relate to short scales for a large-scale panel study, they can be estimated as acceptable for analysis (see e.g., Rammstedt and Beierlein, 2014; Ziegler et al., 2014; Ertl et al., 2020). By applying the Randall program (see Tracey, 1997), we confirmed the hexagonal structure of the dimensions (see Hubert and Arabie, 1987; CI = 0.81; *p* = 0.017; see also Ertl and Hartmann, 2019). Based on the hexagonal structure of the interests, an interest vector comprising all six dimensions was created for each participant according to the suggestions of Prediger (1982) and Eder (1998).

Regarding SOC, a second interest vector for the peer group in a specific subject (e.g., civil engineering, German language) was created. Therefore, interests of all students within a subject were aggregated and the vector was built similarly as for the individual but based on the mean level of the interests of the respective peer group in a specific subject.

For ASP, a third vector was built based on the interest profiles of the occupations listed in the O*Net (2018) database. O*Net contains expert ratings for each profession according to the six interest dimensions. Based on the occupations that students aspired in wave 1, the respective interest vector was assigned to each student. For that, students’ aspirations were first classified according to ISCO-08 (International Labour Office, 2020) classification (e.g., the code 2111 was assigned for *Physicists and Astronomers*). Then, the respective vector from the O*Net data was matched based on the procedure described by Ertl and Hartmann (2019).

Both congruence measures, the SOC as well as the ASP were calculated as the Euclidean distance between the individual and the peer group/aspiration vector as a measure for congruence according to Tracey and Sodano (2013). Notably, low values of this congruence measure indicate a high congruence.

### Persistence and Performance

The NEPS dataset provides a table listing all study episodes of a student (FDZ-LIfBi, 2021b). In this, each study episode was documented, e.g., whether a student started with a BA in computing and then continued with a MA, then the start and end dates of both study programs together with the termination status and the respective grade were documented as two study episodes. If this student concurrently enrolled for biology also this was documented as a further episode. This episode data was applied to extract information about students’ study outcomes. Regarding this, we distinguish two categories: the outcomes of the initial study episode starting at study entry (Initial episode) and, more general, a students’ outcomes within the timeframe of the NEPS panel. We now first look at the initial study episodes starting at study entry. These were analyzed regarding whether students failed (2,143 students) or completed successfully (4,398 students). Moreover, a minor number explicitly mentioned that they didn’t finish these study episodes, or it occurred that they ended this study episode within the first term. Because of the low number of cases, we didn’t include these outcomes in the analyses. Furthermore, the study episodes were still open for about one third of the sample, e.g., due to panel attrition (see Supplementary Material 2).

If students mentioned that they finished a study episode successfully and provided a grade, these grades were taken as performance measure. To ensure comparability, the grades were *z*-standardized within the respective study subject group. This means that we built a comparison group of e.g., students who were successfully finishing a BA in engineering and for this group we built *z*-values indicating how many standard deviations a student’s grade deviated from the group mean. Of note, lower values mean better grades in the German grading system.

In a broader context regarding the general outcomes, we observed that 1,485 of the 4,398 students completed their initial degree and furthermore earned a follow-up degree, e.g., a Master degree after a Bachelor (see Supplementary Material 3). 2,913 students only completed an initial degree. Moreover, we observed that 1,246 students completed another degree but not their initial degree (successful changers). Six hundred and six students filled a reason for dropout questionnaire without earning any degree. Five students previously earned a university degree but not within the NEPS runtime; they will be dropped for further analyses. In conclusion, we don’t have any information about a degree or a dropout for almost 40% of the sample. This number seems reasonable when considering effects of panel attrition and the generally quite high dropout numbers in Germany (see Heublein, 2014).

### Satisfaction

Students’ satisfaction was measured at wave 3 and wave 5 by the scales of Westermann et al. (1996) with the subscales of general study satisfaction (e.g., “I enjoy my degree course”; Cronbach’s α for wave 3: α = 0.880; wave 5: α = 0.890), exhaustion (e.g., “Degree course and other obligations are hard to match”; Cronbach’s α for wave 3: α = 0.776; wave 5: α = 0.773), and satisfaction with study conditions (e.g., “Wishing better study conditions”; Cronbach’s α for wave 3: α = 0.754; wave 5: α = 0.756).

## Results

All analyses were calculated with SPSS 25.0 at the remote NEPS site in Bamberg. Missing values were excluded pairwise for each analysis and therefore Ns are provided for each analysis. Confidence intervals for the correlations were calculated by MPlus 8.2 at the remote NEPS site in Bamberg.

### Congruence Differences for Students With Different Levels of Persistence

The first research question asked how far students with different levels of persistence distinguish regarding their congruence. This endeavor, however, has several framing conditions as all longitudinal studies suffer from panel attrition, implying that members drop out the panel without further information. It may be that they were no more willing to participate but also that they changed their contact information. These students may have abandoned their studies, but they may have also finished their studies and have just left the panel. Therefore, we will consider this group as unknown missing comprising of a part of students missing at random and a part of students missing systematically. We will report their values but exclude this group in the discussion of significant differences. This is different to explicit study dropout when students ended their studies without graduating and gave information about why abandoning their studies.

Regarding the initial study programs (ISP) that started at wave 1, we analyzed mean differences in the congruence levels for students within the different outcome groups. Here we can see that the students who failed showed a significant worse SOC [*F*_Welch(1,3846.545)_ = 44.951; *p* < 0.001; Cohen’s *d* = 0.18] and ASP [*F*_Welch(1,3094.800)_ = 11.504; *p* = 0.001; Cohen’s *d* = 0.11] than their mates that finished successfully. Regarding the general outcomes, we can see significant differences for SOC [*F*_Welch(4,2785.974)_ = 8.999; *p* < 0.001] as well as for ASP [*F*_Welch(4,2067.079)_ = 9.597; *p* < 0.001] with respect to the different outcome categories (see Table 1). *Post Hoc* Tests with Tamhane adjustment revealed that students who were completing their initial degree with or without finishing a follow-up degree showed a significant higher SOC than students that finished after changing their subject or that explicitly dropped out (Cohen’s *d*_max_ = 0.24). With respect to ASP, students who only completed an initial degree showed significantly higher congruence than students who dropped out as well as students that earned a follow up-degree (Cohen’s *d*_*max*_ = 0.25).

**TABLE 1:** Means for social congruence (SOC) and aspirational congruence (ASP) for all students (Total) and separation of male and female students with respect to their initial study program (ISP) and general study outcomes.

Congruence	Total		Male students		Female Students	
ISP outcome	SOC	ASP	SOC	ASP	SOC	ASP
ISP failed	0.372	0.846	0.370	0.920	0.373	0.797
ISP success	0.338	0.812	0.337	0.901	0.339	0.758
**Gen. outcome**						
Open group	0.344	0.818	0.345	0.905	0.344	0.762
Explicit dropout	0.381	0.873	0.365	0.924	0.396	0.830
Successful changers	0.359	0.826	0.353	0.908	0.362	0.782
Initial degree only	0.339	0.793	0.337	0.884	0.340	0.748
Initial degree + follow up	0.338	0.854	0.337	0.930	0.339	0.789

*Lower values indicate higher congruence. N_ISP failed/SOC_ = 2,139; N_ISP failed/ASP_ = 1,646; N_ISP success/SOC_ = 4,393; N_ISP success/ASP_ = 3,303; N_Open Group/SOC_ = 3,969; N_Open Group/ASP_ = 3,051; N_Explicit dropout/SOC_ = 605; N_Explicit dropout/ASP_ = 448; N_Successful changers/SOC_ = 1,243; N_Successful changers/ASP_ = 997; N_Initial degree only/SOC_ = 2,908; N_Initial degree only/ASP_ = 2,299; N_Initial degree + follow up/SOC_ = 1,485; N_Initial degree + follow up/ASP_ = 1,004. The group of ISP success is separated into the groups of Initial degree only and Initial degree + follow up in the lower part of the table. See Supplementary Materials 2, 3 for gender distributions within groups and confidence intervals.*

When investigating gender specifically (RQ1a), the results of the ISP that started at wave 1 showed a lower SOC for male [*F*_Welch(1,1774.717)_ = 19.333; *p* < 0.001; Cohen’s *d* = 0.18] and female students [*F*_Welch(1,2083.846)_ = 25.664; *p* < 0.001; Cohen’s *d* = 0.19] who failed. For ASP, we did not observe an effect for male students (*p* = 0.227) but for female students [*F*_Welch(1,1821.894)_ = 9.684; *p* < 0.01; Cohen’s *d* = 0.12]. Regarding general outcomes, the significant differences disappear for male students (*p* = 0.117/0.141) while they hold for female students for SOC [*F*_Welch(4,1476.769)_ = 7.105; *p* < 0.001] as well as ASP [*F*_Welch(4,1159.132)_ = 4.634; *p* = 0.001]. Again, female students who explicitly dropped out showed a significant worse SOC than female students that completed their initial degree program with or without follow-up (Cohen’s *d*_*max*_ = 0.32). With respect to ASP, we again could only see that female students that completed their initial degree showed a better congruence than female students that dropped out (Cohen’s *d*_*max*_ = 0.27).

Regarding RQ1b that distinguishes the different persistence levels with respect to gender and subject area, we would get a quite extensive table. Therefore, we keep all the congruence values including confidence intervals in the Supplementary Material 4 and just present the *p*-values in Table 2. Besides indicating areas where significant differences occur, these *p*-values give also insights in the areas that are far away from significant differences, which means that the not-significance may be not caused by a decreasing sample size but rather by a non-existence of an effect in the context of a certain area.

**TABLE 2:** *p*-values for differences with respect to social congruence (SOC) and aspirational congruence (ASP) for all students (Total) and separate for male and female students for the different subject areas with respect to their initial study program (ISP) and general study outcomes.

*p-values*	Total		Male students		Female Students	
ISP outcome	SOC	ASP	SOC	ASP	SOC	ASP
STEM-L	0.000***	0.831	0.003**	0.473	0.001**	0.414
STEM-M	0.000***	0.118	0.003**	0.205	0.001**	0.672
Med	0.965	0.344	0.407	0.998	0.644	0.360
Eco	0.053	0.841	0.296	0.550	0.096	0.854
Edu	0.432	0.900	0.386	0.769	0.551	0.826
Lang	0.170	0.011*	0.697	0.416	0.218	0.005**
**Gen. outcome**						
STEM-L	0.101	0.350	0.475	0.076	0.192	0.017*
STEM-M	0.003**	0.263	0.070	0.000***	0.025*	0.308
Med	0.808	0.350	0.750	0.797	0.677	0.562
Eco	0.631	0.551	0.407	0.606	0.152	0.809
Edu	0.794	0.917	0.521	0.955	0.859	0.889
Lang	0.016*	0.230	0.826	0.292	0.023*	0.057

*Because several sub-samples show heterogeneous variances, we will generally report p-values of differences for a robust test of equality means (Welch). Means, SDs, confidence intervals and Ns for the respective subgroups could be found in Supplementary Material 4.*

*p < 0.05; **p < 0.01; ***p < 0.001.

Looking now more detailed into Table 2, we see the differences disappearing for about the half of the subject areas. The *p*-values shown in Table 2 indicate furthermore that most of the missing significances can be explained by non-existent effects rather than by a shrinking sample size. This applies especially for the areas of medicine, economics, and education. When first examining the outcomes of the initial studies, we can see that students in both STEM areas, males as well as females, are highly reliant on SOC with students passing having a significantly higher congruence than students failing (STEM-L: Total Cohen’s *d* = 0.22; male *d* = 0.17; female *d* = 0.37; STEM-M: Total Cohen’s *d* = 0.23; male *d* = 0.25; female *d* = 0.21). For the ASP, there was only one significant effect for the languages (Cohen’s *d* = 0.15), and this is also primarily going back to the female students (Cohen’s *d* = 0.19).

Regarding general outcomes, we still have the effect for STEM-M indicating that dropout students show lower SOC than students that finish their initial degree with follow-up (Cohen’s *d* = 0.30). When separating this for male and female students, we can observe that the significance levels shrink in a way that for male students the differences are only on a tendency level. For female students we had the phenomenon that, although the overall ANOVA indicates significant differences, the *Post Hoc* was not able to assign these to specific group differences. Of note, male students in STEM-M that completed their initial degree program and a follow up showed better ASP than students dropping out (Cohen’s *d* = 0.47). For the languages, students with explicit dropout display lower SOC than students that complete their initial degree and a follow up study (Cohen’s *d* = 0.39). This, however, is particularly demonstrated by the female students (Cohen’s *d* = 0.40). Finally, female students in STEM-L that proceed into a follow-up study exhibit higher congruence than those who do not continue their education (Cohen’s *d* = 0.54).

### Correlations of Congruence and Performance

Looking now at performance (RQ2), we analyze the correlations between congruence and the performance measure study grade. Regarding this we can see that SOC was not related to this measure, neither for the total sample nor for males or females separately (see Table 3). ASP showed only marginal, albeit significant, correlations with the final grades – for the whole sample as well as for male and for female students (RQ2a). Notably, lower values mean better outcomes for both congruence and grades. Therefore, positive correlations indicate that a better fit goes along with a better grade. Looking at the confidence intervals in Supplementary Material 6, we cannot confirm significant differences between male and female students.

**TABLE 3:** Correlations of social congruence (SOC) and aspirational congruence (ASP) with grades for all students (Total) and separation of male and female students.

	Total		Male students		Female Students	
	SOC	ASP	SOC	ASP	SOC	ASP
Final grade (*r*)	0.025	0.061**	0.011	0.071*	0.035	0.056*
*N*	4220	3159	1745	1182	2475	1977

*p < 0.05; **p < 0.01.

When examining different subject areas (RQ2b), we can observe that some correlations were not significant (see Table 4). The remaining, however, increased their sizes. Thus, we could observe a correlation pattern for the final grades indicating that the relation between congruence and performance depended on the subject area and gender. ASP showed stable correlations with the grades for STEM-M for the whole group as well as for male and female students. Regarding languages, the total effect was marginal while male students showed a notable effect, and for education, there was only an effect for the total sample rather than for male and female students in detail. Several other correlations showed values greater than 0.1 but were not significant. Thus, we did not interpret them. With regard to SOC, there were no significant effects at all. Overall, the correlation patterns that emerged when different subject areas are distinguished are quite diverse and simultaneously appear to be specific for some measures (see Supplementary Material 6).

**TABLE 4:** Correlations of social congruence (SOC) and aspirational congruence (ASP) with grades for all students (Total) and separate for male and female students for the different subject areas.

Correlations	Total		Male students		Female students	
Final grade	SOC	ASP	SOC	ASP	SOC	ASP
STEM-L	0.005	0.036	0.019	0.012	–0.059	0.136
STEM-M	0.002	0.127**	–0.010	0.161*	0.008	0.103*
Med	–0.019	0.100	–0.176	0.166	0.053	0.071
Eco	0.064	0.033	0.032	0.073	0.083	0.003
Edu	0.064	0.120*	0.211	0.209	0.054	0.114
Lang	0.049	0.079*	0.033	0.215*	0.054	0.070

*Ns and confidence intervals can be found in Supplementary Material 6.*

*p < 0.05; **p < 0.01.

### Correlations of Congruence and Satisfaction

When examining the satisfaction variables (RQ3), we analyzed correlations between congruence and satisfaction. For this RQ, there were some significant but altogether marginal correlations^2^. Study satisfaction in general did not correlate with any mode of congruence, neither at wave 3 nor at wave 5 (see Table 5). An exception could be male students at wave 3 where a better congruence went along with a higher study satisfaction (RQ3a). However, students with a lower congruence felt more exhausted — only the SOC of male students at wave 3 did not correlate. Effects regarding study conditions were quite specific. We generally found that a higher SOC coincided with more satisfaction about the study conditions. This was only applicable for the total sample in wave 3 as well as for the total sample and the male and female sub-samples in wave 5. Regarding ASP, the correlation was the other way around: a higher ASP went along with a lower satisfaction with the study conditions for the total sample and the female sub-sample at wave 3 and wave 5. We could only observe that a better ASP went along with a higher satisfaction with the study conditions for male students in wave 5. Notably, all the correlations, although some were highly significant, were below the Cohen (1988) threshold for small effects. The confidence intervals in Supplementary Material 7, however, indicate significant differences in the correlations between ASP and study conditions between male and female students at wave 3 as well as at wave 5: While a higher congruence was correlated with a higher study satisfaction for male students, this was opposite and significantly different for female students.

**TABLE 5:** Correlations between social congruence (SOC) and aspirational congruence (ASP) and satisfaction measures for all students (Total) and separate for male and female students.

Correlations	Total		Male students		Female students	
W3	SOC	ASP	SOC	ASP	SOC	ASP
Study satisfaction	–0.008	–0.008	–0.025	–0.043*	0.003	0.007
Exhausted	0.041***	0.041**	0.022	0.062**	0.054***	0.048**
Study conditions	–0.026*	0.053***	–0.029	–0.034	–0.024	0.050**
**W5**						
Study satisfaction	–0.018	–0.005	–0.015	–0.040	–0.021	0.013
Exhausted	0.067***	0.049***	0.048**	0.078***	0.080***	0.061***
Study conditions	–0.048***	0.053**	–0.042*	–0.047*	–0.056***	0.054**

*Lower values indicate higher congruence.*

*p < 0.05; **p < 0.01; ***p < 0.001.

Regarding the subject areas (RQ3b), many of the correlations disappeared (Table 6 summarizes areas with significant differences). On the significance level, we can observe that most of the significant correlations between either mode of congruence and the sub-scales of satisfaction could be found in both STEM domains. Yet, when focusing on the correlation sizes, we noticed that all of them were below Cohen’s (1988) threshold of 0.1 – especially for wave 3. Regarding the STEM subjects, this also applies to wave 5. In wave 5, however, we found a small correlation between SOC and exhaustion for medicine (*r* = 0.111; see Supplementary Material 5) and for economics (*r* = 0.105) between ASP and this dimension – both indicating that students with a better congruence are less exhausted. Thus, although most significances could be found in the area of STEM, only exhaustion at wave 5 showed small effects with congruence for medicine (social) and economics (aspirational).

**TABLE 6:** Areas with significant differences for the correlations between social congruence (SOC) and aspirational congruence (ASP) and satisfaction measures for all students and separate for the different study areas.

W3	STEM-L	STEM-M	Med	Eco	Edu	Lang
Study satisfaction	–/–	–/*	–/–	–/–	*/–	–/–
Exhausted	–/*	–/–	–/–	–/–	–/–	–/–
Study conditions	*/**	–/–	–/–	–/–	–/–	*/–
**W5**						
Study satisfaction	–/–	–/–	–/–	–/–	–/–	–/–
Exhausted	**/–	**/–	*/–	–/*	–/–	–/–
Study conditions	*/**	**/*	–/–	*/–	–/–	–/–

*p < 0.05; **p < 0.01.

Separating these down into gender, we can detect that there were several significant correlations for male students for STEM-L – although all below Cohen’s (1988) threshold of 0.1. Yet, there was one small significant effect for female students: in wave 3, a better ASP was correlated with more satisfaction with the study conditions in wave 5 (*r* = –0.109). For STEM-M there were significant correlations only for female students; however, only one notable effect indicated that a higher congruence went along with more satisfaction with the study conditions (*r* = –0.147; see Supplementary Material 5). For medicine, there were no significant correlations except for the female students in wave 5 indicating that students with a higher SOC felt less exhausted (*r* = 0.137). Male students of economics with a better congruence also felt a higher study satisfaction at wave 3 (*r* = –0.172) as well as less exhaustion at wave 5 (*r* = 0.165). Their female classmates showed two significant correlations, however, with marginal effect sizes. In education, no significant effects were found and in the languages, there was only one with a marginal effect size.

## Discussion

In line with Holland’s (1997) theory of occupational choice and previous research (Allen and Robbins, 2008; Nye et al., 2012; Tracey et al., 2012; Le et al., 2014; Le and Robbins, 2016; Nguyen et al., 2016; Kim and Beier, 2020), we found higher interest congruence (i.e., lower Euclidean distance) for students who persisted in their areas and finished their studies successfully. Thus, hypothesis 1 could be confirmed. The effects (differences) of SOC were overall more distinctive than the ones of ASP – especially for male students who didn’t show significant effects regarding ASP. These overall differences can be broken down to specific subject areas. Especially for STEM areas, SOC is associated with finishing the studies successfully. In addition, our findings regarding academic performance measures (esp. grades) are in line with the results of recent research (Allen and Robbins, 2010; Tracey et al., 2012; Nye et al., 2018) and meta-analytic findings (Nye et al., 2012) since congruence was substantially related to students’ grades. Therefore, hypothesis 2 could also be confirmed. In addition, the results of our study indicate interaction effects as some effects are different for male and female students in different subject areas confirming the conclusions of previous research that the relation between congruence and academic performance is complex and that discipline-unspecific analyzes are insufficient (Nguyen et al., 2016). As with previous research (Assouline and Meir, 1987; Tranberg et al., 1993; Tsabari et al., 2005), the effects of congruence on academic satisfaction indicators were only marginal and weaker than those on persistence and performance. In this regard, having chosen an academic area and aspiring an occupation that fits one’s vocational interests has less impact on being satisfied with the study conditions and with the study in general; however, it has more impact on getting better grades. The stronger impact of congruence on performance than on satisfaction is striking since it not only appears in the academic context but also in the occupational (Hoff et al., 2020).

### Distinctive Effects of Congruence

Reflecting on our study’s outcomes, we can see distinctive effects of social and aspirational congruence. While SOC was more important for students’ persistence, ASP was rather important for students’ performance. We would interpret these differences that students who feel a high congruence with their study mates are more inclined to persist within their studies while students that are more identified with their later job aspiration show more performance. The latter effect may be even stronger as ASP could only be evaluated for students that provided an occupational aspiration at study entry. Thus, looking at our study outcomes, the two types of congruence are rather conceptually different aspects of the person-environment fit than equivalent operationalizations of the same construct (c.f. Kristof-Brown et al., 2005; Su et al., 2015). Since SOC indicates the similarity to the other people in a study subject it may considerably impact the current feeling of belonging, while ASP may be more related to one’s future goals and may therefore lead to put the focus on the need to perform in order to reach the aspired occupation.

### Distinctive Effects for Study Area and Gender

Aside from the differences between the two kinds of congruence, there are area specific and gender differences that are partially responsible for these overall effects. Regarding the gender and subject area, we could see many effects disappearing far beyond significance levels for several areas while the remaining effects increased their effect sizes. This, again, indicates the need for gender and discipline-specific analyses (see e.g., Nguyen et al., 2016).

Person-environment fit theories such as Holland’s theory of vocational choice claim that, in general, the fit between a person and an environment is associated with favorable outcomes. The results of the current study indicate that this general statement can be viewed critically insofar as personal factors such as gender can be related to contextual factors such as sociostructural barriers that influence the effect of congruence (Lent, 2013). In the current study, for STEM subjects with its characteristics, congruence seems to be a more important factor than for other subjects. Considering the big efforts for motivating students studying STEM as well as the high dropout rates, it appears that the study characteristics of STEM studies depend much more on congruence than other subject areas. The reason for this could be that for females in STEM – and especially in STEM-L – a good fit to the aspired occupation is especially important in order to be able and willing to overcome barriers such as gender stereotypes or low self-efficacy (Heilman et al., 2004; Le et al., 2014). In this regard, an aspired occupation can serve as a positive outcome expectation influencing performance outcomes (Lent, 2013). However, females still rarely choose STEM areas that are male labeled as vocational aspirations (OECD, 2013). According to Gottfredson (2005), occupations that do not correspond to one’s own self-concept due to the wrong sex type are already excluded at the age of 6–8 years, which is why appropriate interventions should be started early. This includes the assumption that the effect of gender on the development of vocational interests and aspirations is mediated by socially constructed processes (Lent et al., 1994).

Furthermore, differences in the SOC can be conveyed to the research on the sense of belonging that is especially a challenge for female students in STEM to obtain (see e.g., Deiglmayr et al., 2019; Höhne and Zander, 2019).

Notably, comparable effects may take place in the languages that show an underrepresentation of male students. In closing, the correlations between congruence and exhaustion at wave 5 may indicate specific assessment situations in medicine and economics at this point in time.

### Consequences for Science, Technology, Engineering, Mathematics Interventions

The results have several implications for initiatives for raising young peoples’ interest in STEM. First of all, interest congruence is an important factor for study outcomes, persistence as well as performance, in STEM. Therefore, interest assessment and congruence evaluation, e.g., by the Explorix (Jörin Fux et al., 2003), the SDS (Holland et al., 1973), or the O*Net (2018) can be an important starting point for selecting appropriate participants for interventions in two ways: They help to identify participants with a structure of vocational interests congruent to STEM that, however, are not yet motivated for going into STEM. This might be a promising target group for interventions as they also have good chances for persisting in STEM. On the other hand, students with a low congruence in interest assessments may be better counseled toward different pathways as their chances for persisting STEM are notably lower.

The second aspect relates to the different characteristics of congruence. Although ASP is most important for performance, SOC is the key to persistence. Hands-on activities in science labs (e.g., Paechter et al., 2006) or girls’ days etc. seem rather to support ASP and students’ identification with specific work practices. Such measures seem to motivate students for their later job and rather for performing well in STEM studies. However, such measures tend rather not to cover social aspects like belonging uncertainty (e.g., Deiglmayr et al., 2019; Höhne and Zander, 2019) or stereotypical perspectives about persons working in STEM like Sáinz et al. (2019) report. Thus, initiatives for raising female students’ interests in STEM should also focus the social aspect, especially for the STEM-L area that shows a notable under-representation of female students. Here it seems worth thinking about structuring courses toward groups with similar interest profiles for supporting persistence or at least to provide mentoring and/or coaching programs (e.g., Stein, 2013; Kricorian et al., 2020).

The third aspect relates to the perception of being exhausted by the studies that correlates with congruence for STEM areas. These correlations may relate to perceptions of having to be brilliant for being successful in STEM (e.g., Kessels, 2015; Deiglmayr et al., 2019). Stereotypes about the required “brilliance” that is necessary for succeeding in STEM, together with high failure and dropout rates in STEM (see Heublein, 2014) may open a vicious cycle for students with a lower self-concept for STEM. They may invest more effort as they would need up to being exhausted after few semesters in STEM. Rethinking this exhaustion effect from the background of Expectancy-Value-Theory, one can assume that quite a lot of STEM candidates balance their decision against STEM (see Eccles and Wang, 2016), which further supports the stereotype that rather nerds are in STEM areas (see Ertl et al., 2017; Sáinz et al., 2019). This means, on the long run, to rethink STEM curricula, because initiatives to raise participation in STEM are dependent on the perception that a STEM pathway is an attractive career option.

### Limitations

As with all other large-scale studies, this study also has several limitations. The first relates to the panel design and the respective panel attrition (see Ertl et al., 2020). Regarding the group of students disappearing, it is unclear how far they completed their studies successfully, failed, or dropped out. Looking into their congruence values in Table 1, we could see that these are like the other subgroups. Therefore, we would not expect biasing effects of these students. This applies similarly to students that were so vague in their aspirations that they could neither be classified to a specific occupation nor estimated regarding their ASP. This kind of students may lack in career preparedness (Jaensch et al., 2016) and may be especially present in educational systems of comparably rich countries with low tuition fees like Germany.

Also characteristic for large-scale studies, the current dataset only contained several short scales with comparable low alphas. This issue is intensely discussed in the context of large-scale studies (e.g., Rammstedt and Beierlein, 2014) with Ziegler et al. (2014) concluding that, while less appropriate for individual diagnostics, such short-scales work well for correlational studies like the current one.

Although the strength of this study is to delve deeper into gender- and subject specific differences, this goes along with shrinking sample sizes as well as heterogeneous group sizes for subject areas. This required the use of robust measures that also implied a loss of statistical power. Considering, however, the subject-specific results particularly in Table 2, we hope we were able to demonstrate, by providing the *p-*values, that the non-significances mainly indicate non-effects rather than missing statistical power.

Like this, the study was able to report several significant correlations below the Cohen (1988) threshold of *r* < 0.1. While Cohen (1988) estimates them as marginal or “zero” correlations, and Hattie (2009) postulates only effect sizes larger than 0.4 as desired effects, Funder and Ozer (2019) discuss the value of small and very small effects when they are generalized. This perspective has consequences when considering the almost three Million students and the half Million new students each year in Germany^3^. Although e.g., the correlations between congruence and exhaustion are around *r* = 0.05 which would traditionally mean only 0.25% explained variance, Funder and Ozer (2019) would argue that exhaustion is notably influenced by a missing congruence for several thousand students. We therefore also interpreted several of the low correlations, however, quite cautiously.

## Conclusion

In our study we found evidence for differences between social and aspirational congruence as well as evidence for differences with respect to gender and subject area. Thus, our study supports the conclusion of Nguyen et al. (2016) that discipline-specific approaches are necessary when analyzing factors for university success and retention. Notably, our results also suggest that gender is an important factor to be considered.

The STEM areas were a specific focus of this study and, in comparison with other study areas, we found that congruence, social as well as aspirational, is especially important in these areas. The study could observe several significant or highly significant yet marginal correlations between congruence and outcome measures. These direct future research toward more detailed analyses including further mediating variables. This aligns well with, e.g., Tracey (2007) who suggested looking deeper into moderators or Kieffer et al. (2004) who proposed applying more complex models. The differences which we found between the subject areas, however, indicate that the respective models might be different and consequently include different variables for the subject areas as well as for gender. Especially for female students in STEM, not only interest congruence but also role congruence may be important factors to consider (see Yang and Barth, 2015). While this study aimed at contextualizing the effects of congruence for a broad range of subject areas, follow-up mediation analyses might only be able to compare models for two or three different subject areas.

Our results also opened conclusions for career counseling and initiatives for raising young people’s interest in STEM. Primarily, congruence is an appropriate predictor for study persistence and performance; therefore, it could help to support students’ career decisions as well as the selection of participants for STEM programs. However, congruence is for several subjects more important than for other ones, and thus, career counseling should consider that congruence may be less important for some areas (see also Tracey et al., 2012, p. 48). Further research should therefore have a closer look at environmental constraints that contribute to these differences. Our study made a step forward to this approach by distinguishing SOC (fit with the study group) and ASP (fit with the profession aspired) and found differences in the effects of both. The effects of SOC on persistence especially points toward the risk of self-stabilizing systems: When students fit in better with their classmates, they have higher chance of continuing their study; consequently, study areas homogenize. This may especially apply in areas in which students are underrepresented and have a lower sense of belonging like e.g., female students in STEM-L (see Deiglmayr et al., 2019; Höhne and Zander, 2019). Thus, one should consider that interest assessments may propagate gender differences in occupations – especially in non-traditional ones like Ludwikowski et al. (2019) discuss. Career advisers should be sensitized for such issues, and advice set engines, like Schelfhout et al. (2019a) describe, should be programmed, respectively.

## Data Availability Statement

Publicly available datasets were analyzed in this study. This data can be found here: https://dx.doi.org/10.5157/NEPS:SC5:14.0.0.

## Ethics Statement

The analyses of this manuscript are secondary analyses of data published previously (Blossfeld et al., 2011). Data sources used for the analyses were the cohort of first year students (doi: 10.5157/NEPS:SC5:14.0.0) of the German National Educational Panel Study (Blossfeld et al., 2011). All students from this cohort gave informed consent to participate in the panel by providing their phone number for being contacted for telephone interviews after being informed about the purposes of the study. Specific information about the recruitment process can be found in the field report of the study (Steinwede and Aust, 2012). All data analyses were performed via a remote terminal (RemoteNEPS) at the LIfBi in Bamberg, Germany that provided a controlled privacy environment for data access. Furthermore, an ethics approval for the analyses was obtained by the local ethics committee.

## Author Contributions

All the authors contributed to the study conception and design, commented on previous versions of the manuscript, and read and approved the final manuscript. BE and AW performed the material preparation and data analysis. BE and FH wrote the first draft of the manuscript.

## Conflict of Interest

The authors declare that the research was conducted in the absence of any commercial or financial relationships that could be construed as a potential conflict of interest.

## Publisher’s Note

All claims expressed in this article are solely those of the authors and do not necessarily represent those of their affiliated organizations, or those of the publisher, the editors and the reviewers. Any product that may be evaluated in this article, or claim that may be made by its manufacturer, is not guaranteed or endorsed by the publisher.

## Footnotes

1 More information on students’ reasons for dropout could be found at Mouton et al. (2020).

2 When interpreting these effect sizes, we followed Cohen’s (1988) well-known suggestion that effects smaller than 0.1 are rather negligible.

3
https://www.destatis.de/DE/Presse/Pressemitteilungen/2021/11/PD21_538_21.html;jsessionid=297FC0FD90B067F4E3C4F430121BDCFC.live711
