# Supplementary material for: Impact of Interest Congruence on Study Outcomes
Source: Front Psychol. 2022 Mar 4;13:816620. doi: 10.3389/fpsyg.2022.816620 (PMC8931396; doi:10.3389/fpsyg.2022.816620)
Supplement: Supplementary file 1 [file Data_Sheet_1.PDF]

Supplementary Material

1 Supplement 1: Sample of the study by gender and study area

		Gender		
		male	female	Total
Study area	STEM-L	2344	635	2979
	STEM-M	840	1617	2457
	MED	160	338	498
	ECO	509	860	1369
	EDU	75	648	723
	Lang	341	1859	2200
Total		4269	5957	10226

2 Supplement 2: Study outcomes of the initial study episode by gender

		Gender		
		male	female	Total
Initial episode 1 termination status	failed	922	1221	2143
	successful finished	1815	2583	4398
Total		2737	3804	6541

3 Supplement 3: General study outcomes by gender

		Gender		
		male	female	Total
Finishing status of degrees	No Degree	1680	2291	3971
	Explicit dropout	297	309	606
	Successful changers	477	769	1246
	Initial degree only only	1078	1835	2913
	Initial degree + follow up	737	748	1485
Total		4269	5952	10221

4 Supplement 4: Ns, Means, standard deviations for Tables 1 and 2

4.1 Supplement 4a: Means and standard deviations for Table 1: Initial Study Episode - Total

		N	Mean	Std. Deviation	Std. Error	95% Confidence Interval for Mean	
						Lower Bound	Upper Bound
Social congruence	failed	2139	0.3720	0.19661	0.00425	0.3636	0.3803
	successful finished	4393	0.3384	0.17598	0.00266	0.3332	0.3436
	Total	6532	0.3494	0.18366	0.00227	0.3449	0.3538
Aspirational congruence	failed	1646	0.8455	0.33911	0.00836	0.8291	0.8619
	successful finished	3303	0.8115	0.31674	0.00551	0.8007	0.8223
	Total	4949	0.8228	0.32471	0.00462	0.8137	0.8318

4.2 Supplement 4b: Means and standard deviations for Table 1: General study outcome - Total

		N	Mean	Std. Deviation	Std. Error	95% Confidence Interval for Mean	
						Lower Bound	Upper Bound
Social congruence	No Degree	3969	0.3440	0.18475	0.00293	0.3382	0.3497
	Explicit dropout	605	0.3808	0.19653	0.00799	0.3651	0.3965
	Successful changers	1243	0.3585	0.19038	0.00540	0.3479	0.3691
	Initial degree	2908	0.3387	0.17612	0.00327	0.3323	0.3451
	Initial Degree + follow up	1485	0.3378	0.17577	0.00456	0.3288	0.3467
	Total	10210	0.3455	0.18276	0.00181	0.3420	0.3491
Aspirational congruence	No Degree	3051	0.8175	0.33105	0.00599	0.8057	0.8292
	Explicit dropout	448	0.8728	0.35522	0.01678	0.8398	0.9058
	Successful changers	997	0.8256	0.31911	0.01011	0.8057	0.8454
	Initial degree	2299	0.7930	0.31349	0.00654	0.7802	0.8058
	Initial Degree + follow up	1004	0.8538	0.32022	0.01011	0.8340	0.8737
	Total	7799	0.8192	0.32524	0.00368	0.8119	0.8264

4.3 Supplement 4c: Means and standard deviations for Table 1: Initial Study Episode – Male and Female Students

Gender			N	Mean	Std. Deviation	Std. Error	95% Confidence Interval for Mean	
							Lower Bound	Upper Bound
male	Social congruence	failed	921	0.3701	0.18998	0.00626	0.3579	0.3824
		successful finished	1813	0.3369	0.18124	0.00426	0.3285	0.3452
		Total	2734	0.3481	0.18487	0.00354	0.3411	0.3550
	Aspirational congruence	failed	647	0.9203	0.33609	0.01321	0.8944	0.9462
		successful finished	1233	0.9009	0.31810	0.00906	0.8832	0.9187
		Total	1880	0.9076	0.32444	0.00748	0.8929	0.9223
female	Social congruence	failed	1218	0.3733	0.20154	0.00577	0.3620	0.3847
		successful finished	2580	0.3394	0.17222	0.00339	0.3328	0.3461
		Total	3798	0.3503	0.18280	0.00297	0.3445	0.3561
	Aspirational congruence	failed	999	0.7970	0.33235	0.01052	0.7764	0.8176
		successful finished	2070	0.7582	0.30373	0.00668	0.7451	0.7713
		Total	3069	0.7708	0.31380	0.00566	0.7597	0.7820

4.4 Supplement 4d: Means and standard deviations for Table 1: General study outcome - Male and Female Students

Gender			N	Mean	Std. Deviation	Std. Error	95% Confidence Interval for Mean	
							Lower Bound	Upper Bound
male	Social congruence	No Degree	1679	0.3446	0.18555	0.00453	0.3357	0.3535
		Explicit dropout	296	0.3646	0.18916	0.01099	0.3430	0.3862
		Successful changers	477	0.3527	0.18102	0.00829	0.3365	0.3690
		Initial degree	1076	0.3371	0.18083	0.00551	0.3263	0.3479
		Initial Degree + follow up	737	0.3365	0.18196	0.00670	0.3234	0.3497
		Total	4265	0.3436	0.18358	0.00281	0.3381	0.3491
	Aspirational congruence	No Degree	1184	0.9047	0.33006	0.00959	0.8859	0.9235
		Explicit dropout	204	0.9243	0.34903	0.02444	0.8761	0.9725
		Successful changers	345	0.9076	0.31513	0.01697	0.8742	0.9410
		Initial degree	769	0.8836	0.31851	0.01149	0.8611	0.9062
		Initial Degree + follow up	464	0.9296	0.31567	0.01465	0.9008	0.9584
		Total	2966	0.9048	0.32466	0.00596	0.8931	0.9165
female	Social congruence	No Degree	2290	0.3435	0.18420	0.00385	0.3360	0.3511
		Explicit dropout	309	0.3964	0.20243	0.01152	0.3737	0.4190
		Successful changers	766	0.3621	0.19601	0.00708	0.3482	0.3760
		Initial degree	1832	0.3396	0.17334	0.00405	0.3316	0.3475
		Initial Degree + follow up	748	0.3390	0.16955	0.00620	0.3269	0.3512
		Total	5945	0.3469	0.18218	0.00236	0.3423	0.3515
	Aspirational congruence	No Degree	1867	0.7622	0.31965	0.00740	0.7477	0.7767
		Explicit dropout	244	0.8298	0.35532	0.02275	0.7850	0.8746
		Successful changers	652	0.7822	0.31284	0.01225	0.7581	0.8062
		Initial degree	1530	0.7475	0.30090	0.00769	0.7324	0.7626
		Initial Degree + follow up	540	0.7887	0.30987	0.01333	0.7625	0.8149
		Total	4833	0.7666	0.31427	0.00452	0.7577	0.7755

4.5 Supplement 4e: Means and standard deviations for Table 2: students' initial episode – Whole group

Study area			N	Mean	Std. Deviation	Std. Error	95% Confidence Interval for Mean	
							Lower Bound	Upper Bound
STEM-L	Social congruence	failed	613	0.3804	0.20461	0.00826	0.3642	0.3967
		successful finished	1279	0.3394	0.18230	0.00510	0.3294	0.3494
		Total	1892	0.3527	0.19073	0.00438	0.3441	0.3613
	Aspirational congruence	failed	386	0.9442	0.34526	0.01757	0.9096	0.9787
		successful finished	762	0.9486	0.31436	0.01139	0.9263	0.9710
		Total	1148	0.9471	0.32493	0.00959	0.9283	0.9659
STEM-M	Social congruence	failed	665	0.3895	0.19620	0.00761	0.3746	0.4045
		successful finished	1031	0.3475	0.17421	0.00543	0.3369	0.3582
		Total	1696	0.3640	0.18424	0.00447	0.3552	0.3728
	Aspirational congruence	failed	551	0.9266	0.32460	0.01383	0.8994	0.9537
		successful finished	829	0.8999	0.28923	0.01005	0.8802	0.9196
		Total	1380	0.9105	0.30401	0.00818	0.8945	0.9266
MED	Social congruence	failed	38	0.3339	0.16812	0.02727	0.2786	0.3891
		successful finished	255	0.3326	0.17205	0.01077	0.3114	0.3538
		Total	293	0.3327	0.17126	0.01001	0.3130	0.3524
	Aspirational congruence	failed	32	0.7420	0.30388	0.05372	0.6325	0.8516
		successful finished	249	0.6885	0.24236	0.01536	0.6583	0.7188
		Total	281	0.6946	0.25007	0.01492	0.6652	0.7240
ECO	Social congruence	failed	187	0.3754	0.20271	0.01482	0.3462	0.4047
		successful finished	638	0.3435	0.17932	0.00710	0.3295	0.3574
		Total	825	0.3507	0.18523	0.00645	0.3380	0.3634
	Aspirational congruence	failed	116	0.8486	0.32480	0.03016	0.7889	0.9083
		successful finished	397	0.8418	0.31298	0.01571	0.8109	0.8727

		Total	513	0.8433	0.31538	0.01392	0.8160	0.8707
EDU	Social congruence	failed	87	0.3305	0.19117	0.02050	0.2898	0.3713
		successful finished	411	0.3131	0.16519	0.00815	0.2971	0.3292
		Total	498	0.3162	0.16993	0.00761	0.3012	0.3311
	Aspirational congruence	failed	67	0.6412	0.31938	0.03902	0.5633	0.7191
		successful finished	331	0.6466	0.32215	0.01771	0.6118	0.6814
		Total	398	0.6457	0.32129	0.01610	0.6140	0.6774
Lang	Social congruence	failed	549	0.3493	0.18567	0.00792	0.3337	0.3649
		successful finished	779	0.3356	0.17088	0.00612	0.3235	0.3476
		Total	1328	0.3412	0.17721	0.00486	0.3317	0.3508
	Aspirational congruence	failed	494	0.7115	0.29948	0.01347	0.6850	0.7380
		successful finished	735	0.6692	0.26198	0.00966	0.6502	0.6882
		Total	1229	0.6862	0.27832	0.00794	0.6706	0.7018

4.6 Supplement 4f: Means and standard deviations for Table 2: Male students' initial episode

Study area			N	Mean	Std. Deviation	Std. Error	95% Confidence Interval for Mean	
							Lower Bound	Upper Bound
STEM-L	Social congruence	failed	478	0.3600	0.19025	0.00870	0.3429	0.3771
		successful finished	1007	0.3288	0.18212	0.00574	0.3175	0.3400
		Total	1485	0.3388	0.18529	0.00481	0.3294	0.3482
	Aspirational congruence	failed	296	0.9321	0.33281	0.01934	0.8940	0.9702
		successful finished	613	0.9499	0.30486	0.01231	0.9257	0.9741
		Total	909	0.9441	0.31416	0.01042	0.9237	0.9646
STEM-M	Social congruence	failed	252	0.3965	0.18850	0.01187	0.3731	0.4199
		successful finished	342	0.3507	0.17901	0.00968	0.3316	0.3697
		Total	594	0.3701	0.18434	0.00756	0.3553	0.3850
	Aspirational congruence	failed	203	1.0186	0.31168	0.02188	0.9754	1.0617
		successful finished	252	0.9816	0.30553	0.01925	0.9437	1.0195
		Total	455	0.9981	0.30850	0.01446	0.9696	1.0265
MED	Social congruence	failed	11	0.3180	0.11969	0.03609	0.2376	0.3984
		successful finished	83	0.3532	0.18452	0.02025	0.3129	0.3935
		Total	94	0.3491	0.17802	0.01836	0.3126	0.3855
	Aspirational congruence	failed	8	0.6477	0.18592	0.06573	0.4923	0.8031
		successful finished	81	0.6475	0.25574	0.02842	0.5909	0.7040
		Total	89	0.6475	0.24941	0.02644	0.5950	0.7001
ECO	Social congruence	failed	61	0.3804	0.21057	0.02696	0.3265	0.4343
		successful finished	243	0.3494	0.18556	0.01190	0.3260	0.3729
		Total	304	0.3556	0.19088	0.01095	0.3341	0.3772
	Aspirational congruence	failed	33	0.8842	0.37512	0.06530	0.7512	1.0173
		successful finished	163	0.8421	0.32157	0.02519	0.7923	0.8918

		Total	196	0.8492	0.33053	0.02361	0.8026	0.8957
EDU	Social congruence	failed	11	0.3302	0.14864	0.04482	0.2303	0.4300
		successful finished	39	0.2851	0.14667	0.02349	0.2375	0.3326
		Total	50	0.2950	0.14679	0.02076	0.2533	0.3367
	Aspirational congruence	failed	7	0.6742	0.29034	0.10974	0.4057	0.9427
		successful finished	31	0.6376	0.28005	0.05030	0.5349	0.7404
		Total	38	0.6444	0.27833	0.04515	0.5529	0.7359
Lang	Social congruence	failed	108	0.3572	0.18600	0.01790	0.3217	0.3926
		successful finished	99	0.3474	0.17433	0.01752	0.3126	0.3822
		Total	207	0.3525	0.18014	0.01252	0.3278	0.3772
	Aspirational congruence	failed	100	0.7369	0.30142	0.03014	0.6771	0.7967
		successful finished	93	0.7712	0.28410	0.02946	0.7127	0.8297
		Total	193	0.7534	0.29294	0.02109	0.7118	0.7950

4.7 Supplement 4g: Means and standard deviations for Table 2: Female students' initial episode

Study area			N	Mean	Std. Deviation	Std. Error	95% Confidence Interval for Mean	
							Lower Bound	Upper Bound
STEM-L	Social congruence	failed	135	0.4528	0.23578	0.02029	0.4127	0.4929
		successful finished	272	0.3788	0.17781	0.01078	0.3576	0.4001
		Total	407	0.4034	0.20166	0.01000	0.3837	0.4230
	Aspirational congruence	failed	90	0.9839	0.38267	0.04034	0.9037	1.0640
		successful finished	149	0.9432	0.35183	0.02882	0.8863	1.0002
		Total	239	0.9585	0.36349	0.02351	0.9122	1.0049
STEM-M	Social congruence	failed	413	0.3852	0.20086	0.00988	0.3658	0.4047
		successful finished	689	0.3460	0.17189	0.00655	0.3331	0.3588
		Total	1102	0.3607	0.18418	0.00555	0.3498	0.3716
	Aspirational congruence	failed	348	0.8729	0.32035	0.01717	0.8392	0.9067
		successful finished	577	0.8642	0.27456	0.01143	0.8418	0.8867
		Total	925	0.8675	0.29249	0.00962	0.8486	0.8864
MED	Social congruence	failed	27	0.3403	0.18591	0.03578	0.2668	0.4139
		successful finished	172	0.3226	0.16534	0.01261	0.2977	0.3475
		Total	199	0.3250	0.16788	0.01190	0.3016	0.3485
	Aspirational congruence	failed	24	0.7735	0.33138	0.06764	0.6335	0.9134
		successful finished	168	0.7083	0.23385	0.01804	0.6727	0.7439
		Total	192	0.7164	0.24800	0.01790	0.6811	0.7517
ECO	Social congruence	failed	126	0.3730	0.19961	0.01778	0.3378	0.4082
		successful finished	395	0.3398	0.17551	0.00883	0.3224	0.3572
		Total	521	0.3478	0.18199	0.00797	0.3322	0.3635
	Aspirational congruence	failed	83	0.8344	0.30385	0.03335	0.7681	0.9008
		successful finished	234	0.8416	0.30755	0.02011	0.8020	0.8812

		Total	317	0.8397	0.30612	0.01719	0.8059	0.8736
EDU	Social congruence	failed	76	0.3306	0.19738	0.02264	0.2855	0.3757
		successful finished	372	0.3161	0.16691	0.00865	0.2991	0.3331
		Total	448	0.3185	0.17231	0.00814	0.3025	0.3345
	Aspirational congruence	failed	60	0.6374	0.32464	0.04191	0.5535	0.7213
		successful finished	300	0.6475	0.32659	0.01886	0.6104	0.6846
		Total	360	0.6458	0.32584	0.01717	0.6121	0.6796
Lang	Social congruence	failed	441	0.3474	0.18576	0.00885	0.3300	0.3648
		successful finished	680	0.3338	0.17043	0.00654	0.3210	0.3467
		Total	1121	0.3392	0.17666	0.00528	0.3288	0.3495
	Aspirational congruence	failed	394	0.7051	0.29902	0.01506	0.6755	0.7347
		successful finished	642	0.6544	0.25550	0.01008	0.6346	0.6742
		Total	1036	0.6737	0.27384	0.00851	0.6570	0.6904

4.8 Supplement 4h: Means and standard deviations for Table 2: students' general outcomes – Whole group

							95% Confidence Interval for Mean	
Study area			N	Mean	Std. Deviation	Std. Error	Lower Bound	Upper Bound
STEM-L	Social congruence	No Degree	1149	0.3564	0.18760	0.00553	0.3455	0.3673
		Explicit dropout	210	0.3620	0.19910	0.01374	0.3349	0.3891
		Successful changers	337	0.3626	0.20528	0.01118	0.3406	0.3846
		Initial degree	688	0.3404	0.18906	0.00721	0.3263	0.3546
		Initial Degree + follow up	591	0.3382	0.17425	0.00717	0.3242	0.3523
		Total	2975	0.3502	0.18843	0.00345	0.3434	0.3570
	Aspirational congruence	No Degree	714	0.9684	0.33107	0.01239	0.9441	0.9928
		Explicit dropout	137	0.9273	0.36975	0.03159	0.8648	0.9897
		Successful changers	216	0.9230	0.30155	0.02052	0.8825	0.9634
		Initial degree	414	0.9477	0.32044	0.01575	0.9168	0.9787
		Initial Degree + follow up	348	0.9497	0.30742	0.01648	0.9173	0.9821
		Total	1829	0.9517	0.32406	0.00758	0.9369	0.9666
STEM-M	Social congruence	No Degree	858	0.3568	0.19015	0.00649	0.3440	0.3695
		Explicit dropout	193	0.3970	0.19067	0.01372	0.3699	0.4241
		Successful changers	373	0.3776	0.18336	0.00949	0.3590	0.3963
		Initial degree	630	0.3500	0.17383	0.00693	0.3364	0.3636
		Initial Degree + follow up	401	0.3437	0.17495	0.00874	0.3265	0.3608
		Total	2455	0.3592	0.18311	0.00370	0.3520	0.3665
	Aspirational congruence	No Degree	708	0.8918	0.30410	0.01143	0.8694	0.9143
		Explicit dropout	148	0.9495	0.31767	0.02611	0.8979	1.0011
		Successful changers	332	0.9183	0.31290	0.01717	0.8845	0.9521
		Initial degree	546	0.8962	0.28813	0.01233	0.8720	0.9204
		Initial Degree + follow up	283	0.9070	0.29173	0.01734	0.8729	0.9412

		Total	2017	0.9037	0.30078	0.00670	0.8906	0.9169
MED	Social congruence	No Degree	201	0.3188	0.17762	0.01253	0.2941	0.3435
		Explicit dropout	14	0.3477	0.14772	0.03948	0.2624	0.4330
		Successful changers	27	0.3497	0.16157	0.03109	0.2858	0.4136
		Initial degree	238	0.3306	0.16774	0.01087	0.3092	0.3520
		Initial Degree + follow up	17	0.3602	0.22866	0.05546	0.2426	0.4778
		Total	497	0.3284	0.17292	0.00776	0.3131	0.3436
	Aspirational congruence	No Degree	190	0.6964	0.24147	0.01752	0.6619	0.7310
		Explicit dropout	13	0.8234	0.25769	0.07147	0.6677	0.9791
		Successful changers	23	0.6569	0.33461	0.06977	0.5122	0.8016
		Initial degree	232	0.6931	0.24124	0.01584	0.6619	0.7243
		Initial Degree + follow up	17	0.6260	0.25641	0.06219	0.4942	0.7579
		Total	475	0.6938	0.24780	0.01137	0.6715	0.7162
ECO	Social congruence	No Degree	572	0.3407	0.18668	0.00781	0.3254	0.3561
		Explicit dropout	59	0.3793	0.21379	0.02783	0.3236	0.4350
		Successful changers	95	0.3620	0.19996	0.02052	0.3212	0.4027
		Initial degree	433	0.3447	0.17496	0.00841	0.3282	0.3612
		Initial Degree + follow up	205	0.3408	0.18860	0.01317	0.3149	0.3668
		Total	1364	0.3452	0.18550	0.00502	0.3353	0.3550
	Aspirational congruence	No Degree	352	0.8741	0.33777	0.01800	0.8387	0.9095
		Explicit dropout	38	0.8562	0.35209	0.05712	0.7404	0.9719
		Successful changers	57	0.8094	0.31737	0.04204	0.7252	0.8936
		Initial degree	275	0.8383	0.30931	0.01865	0.8015	0.8750
		Initial Degree + follow up	122	0.8497	0.32225	0.02918	0.7920	0.9075
		Total	844	0.8537	0.32561	0.01121	0.8317	0.8757
EDU	Social congruence	No Degree	233	0.3177	0.16918	0.01108	0.2959	0.3396
		Explicit dropout	15	0.3729	0.23071	0.05957	0.2451	0.5006
		Successful changers	62	0.3049	0.16807	0.02134	0.2622	0.3476

		Initial degree	326	0.3103	0.16454	0.00911	0.2924	0.3283
		Initial Degree + follow up	85	0.3238	0.16819	0.01824	0.2876	0.3601
		Total	721	0.3151	0.16814	0.00626	0.3029	0.3274
	Aspirational congruence	No Degree	200	0.6284	0.29671	0.02098	0.5870	0.6698
		Explicit dropout	10	0.5813	0.37923	0.11992	0.3100	0.8526
		Successful changers	50	0.6624	0.32935	0.04658	0.5688	0.7560
		Initial degree	269	0.6445	0.32246	0.01966	0.6058	0.6832
		Initial Degree + follow up	62	0.6559	0.32327	0.04106	0.5738	0.7380
		Total	591	0.6407	0.31483	0.01295	0.6152	0.6661
Lang	Social congruence	No Degree	956	0.3312	0.17851	0.00577	0.3199	0.3425
		Explicit dropout	114	0.3940	0.19333	0.01811	0.3581	0.4299
		Successful changers	349	0.3433	0.18401	0.00985	0.3239	0.3627
		Initial degree	593	0.3390	0.17215	0.00707	0.3251	0.3528
		Initial Degree + follow up	186	0.3247	0.16677	0.01223	0.3006	0.3488
		Total	2198	0.3379	0.17795	0.00380	0.3305	0.3454
	Aspirational congruence	No Degree	887	0.6827	0.29053	0.00976	0.6636	0.7019
		Explicit dropout	102	0.7296	0.34522	0.03418	0.6618	0.7974
		Successful changers	319	0.7038	0.27706	0.01551	0.6732	0.7343
		Initial degree	563	0.6692	0.25830	0.01089	0.6479	0.6906
		Initial Degree + follow up	172	0.6692	0.27448	0.02093	0.6279	0.7105
		Total	2043	0.6835	0.28176	0.00623	0.6713	0.6957

4.9 Supplement 4i: Means and standard deviations for Table 2: Male students' general outcomes

Study area			N	Mean	Std. Deviation	Std. Error	95% Confidence Interval for Mean	
							Lower Bound	Upper Bound
STEM-L	Social congruence	No Degree	904	0.3426	0.17790	0.00592	0.3310	0.3543
		Explicit dropout	160	0.3402	0.17839	0.01410	0.3124	0.3681
		Successful changers	271	0.3449	0.19248	0.01169	0.3218	0.3679
		Initial degree	542	0.3297	0.18717	0.00804	0.3139	0.3455
		Initial Degree + follow up	465	0.3277	0.17625	0.00817	0.3116	0.3437
		Total	2342	0.3368	0.18151	0.00375	0.3294	0.3441
	Aspirational congruence	No Degree	563	0.9628	0.33056	0.01393	0.9355	0.9902
		Explicit dropout	102	0.9019	0.35140	0.03479	0.8329	0.9709
		Successful changers	174	0.9181	0.29950	0.02271	0.8733	0.9629
		Initial degree	339	0.9284	0.31352	0.01703	0.8949	0.9619
		Initial Degree + follow up	274	0.9765	0.29218	0.01765	0.9418	1.0113
		Total	1452	0.9477	0.31805	0.00835	0.9314	0.9641
STEM-M	Social congruence	No Degree	308	0.3503	0.19018	0.01084	0.3290	0.3716
		Explicit dropout	78	0.4051	0.18348	0.02078	0.3638	0.4465
		Successful changers	112	0.3671	0.17176	0.01623	0.3349	0.3993
		Initial degree	199	0.3628	0.18076	0.01281	0.3375	0.3881
		Initial Degree + follow up	143	0.3338	0.17577	0.01470	0.3047	0.3628
		Total	840	0.3578	0.18306	0.00632	0.3454	0.3702
	Aspirational congruence	No Degree	244	0.9328	0.29717	0.01902	0.8954	0.9703
		Explicit dropout	58	1.0645	0.28877	0.03792	0.9886	1.1404
		Successful changers	97	1.0413	0.29437	0.02989	0.9820	1.1006
		Initial degree	155	1.0198	0.29039	0.02332	0.9737	1.0659
		Initial Degree + follow up	97	0.9204	0.32041	0.03253	0.8558	0.9850
		Total	651	0.9796	0.30201	0.01184	0.9563	1.0028
MED	Social congruence	No Degree	64	0.3167	0.16664	0.02083	0.2751	0.3583

		Explicit dropout	6	0.2931	0.14976	0.06114	0.1360	0.4503
		Successful changers	7	0.3290	0.12083	0.04567	0.2173	0.4408
		Initial degree	75	0.3463	0.16984	0.01961	0.3072	0.3854
		Initial Degree + follow up	8	0.4179	0.29768	0.10524	0.1690	0.6668
		Total	160	0.3353	0.17376	0.01374	0.3081	0.3624
	Aspirational congruence	No Degree	61	0.6558	0.24512	0.03138	0.5930	0.7186
		Explicit dropout	5	0.7357	0.17849	0.07982	0.5141	0.9574
		Successful changers	5	0.5548	0.29237	0.13075	0.1918	0.9178
		Initial degree	73	0.6522	0.25565	0.02992	0.5925	0.7118
		Initial Degree + follow up	8	0.6049	0.27004	0.09547	0.3792	0.8307
		Total	152	0.6507	0.24938	0.02023	0.6107	0.6907
ECO	Social congruence	No Degree	213	0.3444	0.19929	0.01366	0.3175	0.3713
		Explicit dropout	23	0.3727	0.22402	0.04671	0.2759	0.4696
		Successful changers	28	0.3639	0.16785	0.03172	0.2988	0.4289
		Initial degree	155	0.3318	0.16987	0.01364	0.3049	0.3588
		Initial Degree + follow up	88	0.3803	0.20779	0.02215	0.3363	0.4244
		Total	507	0.3492	0.19193	0.00852	0.3324	0.3659
	Aspirational congruence	No Degree	138	0.8735	0.35438	0.03017	0.8139	0.9332
		Explicit dropout	13	0.9123	0.42351	0.11746	0.6564	1.1682
		Successful changers	17	0.7612	0.27681	0.06714	0.6189	0.9035
		Initial degree	107	0.8370	0.29495	0.02851	0.7804	0.8935
		Initial Degree + follow up	56	0.8518	0.36983	0.04942	0.7527	0.9508
		Total	331	0.8538	0.33756	0.01855	0.8173	0.8903
EDU	Social congruence	No Degree	27	0.3561	0.20205	0.03888	0.2762	0.4360
		Explicit dropout	2	0.3477	0.05003	0.03537	-0.1018	0.7971
		Successful changers	7	0.3213	0.15250	0.05764	0.1803	0.4623
		Initial degree	32	0.2770	0.14233	0.02516	0.2257	0.3283

		Initial Degree + follow up	7	0.3220	0.17216	0.06507	0.1628	0.4813
		Total	75	0.3157	0.16861	0.01947	0.2769	0.3545
	Aspirational congruence	No Degree	23	0.6832	0.33271	0.06937	0.5394	0.8271
		Explicit dropout	2	0.6043	0.41298	0.29202	-3.1062	4.3148
		Successful changers	5	0.7260	0.30153	0.13485	0.3516	1.1004
		Initial degree	27	0.6274	0.25273	0.04864	0.5275	0.7274
		Initial Degree + follow up	4	0.7065	0.47268	0.23634	-0.0456	1.4587
		Total	61	0.6610	0.29939	0.03833	0.5843	0.7377
Lang	Social congruence	No Degree	163	0.3537	0.20455	0.01602	0.3221	0.3853
		Explicit dropout	27	0.4022	0.23120	0.04450	0.3108	0.4937
		Successful changers	52	0.3644	0.15784	0.02189	0.3204	0.4083
		Initial degree	73	0.3497	0.17578	0.02057	0.3087	0.3907
		Initial Degree + follow up	26	0.3409	0.17347	0.03402	0.2709	0.4110
		Total	341	0.3573	0.19163	0.01038	0.3369	0.3777
	Aspirational congruence	No Degree	155	0.8079	0.30343	0.02437	0.7598	0.8561
		Explicit dropout	24	0.7533	0.34753	0.07094	0.6066	0.9001
		Successful changers	47	0.7027	0.27646	0.04033	0.6216	0.7839
		Initial degree	68	0.7734	0.29468	0.03574	0.7021	0.8447
		Initial Degree + follow up	25	0.7653	0.25869	0.05174	0.6585	0.8721
		Total	319	0.7776	0.29843	0.01671	0.7448	0.8105

4.10 Supplement 4j: Means and standard deviations for Table R2: Female Students general outcomes

Study area			N	Mean	Std. Deviation	Std. Error	95% Confidence Interval for Mean	
							Lower Bound	Upper Bound
STEM-L	Social congruence	No Degree	245	0.4072	0.21253	0.01358	0.3804	0.4339
		Explicit dropout	50	0.4318	0.24352	0.03444	0.3626	0.5010
		Successful changers	66	0.4355	0.23925	0.02945	0.3767	0.4943
		Initial degree	146	0.3802	0.19138	0.01584	0.3489	0.4115
		Initial Degree + follow up	126	0.3773	0.16141	0.01438	0.3488	0.4057
		Total	633	0.3999	0.20470	0.00814	0.3839	0.4159
	Aspirational congruence	No Degree	151	0.9894	0.33324	0.02712	0.9358	1.0430
		Explicit dropout	35	1.0013	0.41532	0.07020	0.8586	1.1439
		Successful changers	42	0.9431	0.31278	0.04826	0.8456	1.0405
		Initial degree	75	1.0348	0.33871	0.03911	0.9569	1.1128
		Initial Degree + follow up	74	0.8504	0.34250	0.03981	0.7711	0.9298
		Total	377	0.9671	0.34625	0.01783	0.9320	1.0022
STEM-M	Social congruence	No Degree	550	0.3604	0.19021	0.00811	0.3445	0.3763
		Explicit dropout	115	0.3915	0.19600	0.01828	0.3553	0.4277
		Successful changers	261	0.3822	0.18826	0.01165	0.3592	0.4051
		Initial degree	431	0.3440	0.17043	0.00821	0.3279	0.3602
		Initial Degree + follow up	258	0.3491	0.17460	0.01087	0.3277	0.3705
		Total	1615	0.3600	0.18319	0.00456	0.3510	0.3689
	Aspirational congruence	No Degree	464	0.8703	0.30579	0.01420	0.8424	0.8982
		Explicit dropout	90	0.8753	0.31478	0.03318	0.8094	0.9413
		Successful changers	235	0.8675	0.30674	0.02001	0.8281	0.9070
		Initial degree	391	0.8472	0.27246	0.01378	0.8201	0.8743
		Initial Degree + follow up	186	0.9000	0.27624	0.02026	0.8601	0.9400

		Total	1366	0.8676	0.29348	0.00794	0.8520	0.8832
MED	Social congruence	No Degree	137	0.3198	0.18312	0.01564	0.2889	0.3508
		Explicit dropout	8	0.3886	0.14154	0.05004	0.2703	0.5070
		Successful changers	20	0.3570	0.17578	0.03931	0.2747	0.4392
		Initial degree	163	0.3234	0.16679	0.01306	0.2976	0.3492
		Initial Degree + follow up	9	0.3089	0.14403	0.04801	0.1982	0.4196
		Total	337	0.3251	0.17269	0.00941	0.3066	0.3436
	Aspirational congruence	No Degree	129	0.7156	0.23828	0.02098	0.6741	0.7571
		Explicit dropout	8	0.8781	0.29448	0.10411	0.6320	1.1243
		Successful changers	18	0.6852	0.34766	0.08194	0.5123	0.8581
		Initial degree	159	0.7119	0.23275	0.01846	0.6754	0.7483
		Initial Degree + follow up	9	0.6448	0.25854	0.08618	0.4461	0.8436
		Total	323	0.7141	0.24481	0.01362	0.6873	0.7409
ECO	Social congruence	No Degree	359	0.3386	0.17903	0.00945	0.3200	0.3571
		Explicit dropout	36	0.3835	0.21012	0.03502	0.3124	0.4546
		Successful changers	67	0.3612	0.21311	0.02604	0.3092	0.4132
		Initial degree	278	0.3519	0.17763	0.01065	0.3309	0.3729
		Initial Degree + follow up	117	0.3111	0.16762	0.01550	0.2804	0.3418
		Total	857	0.3428	0.18165	0.00621	0.3306	0.3550
	Aspirational congruence	No Degree	214	0.8745	0.32745	0.02238	0.8304	0.9186
		Explicit dropout	25	0.8270	0.31438	0.06288	0.6972	0.9567
		Successful changers	40	0.8299	0.33428	0.05285	0.7230	0.9368
		Initial degree	168	0.8391	0.31899	0.02461	0.7905	0.8877
		Initial Degree + follow up	66	0.8480	0.27852	0.03428	0.7795	0.9165
		Total	513	0.8537	0.31800	0.01404	0.8261	0.8813
EDU	Social congruence	No Degree	206	0.3127	0.16430	0.01145	0.2901	0.3353
		Explicit dropout	13	0.3768	0.24853	0.06893	0.2266	0.5269
		Successful changers	55	0.3028	0.17113	0.02307	0.2565	0.3491

		Initial degree	294	0.3140	0.16659	0.00972	0.2949	0.3331
		Initial Degree + follow up	78	0.3240	0.16896	0.01913	0.2859	0.3621
		Total	646	0.3151	0.16822	0.00662	0.3021	0.3281
	Aspirational congruence	No Degree	177	0.6213	0.29200	0.02195	0.5779	0.6646
		Explicit dropout	8	0.5756	0.40044	0.14158	0.2408	0.9104
		Successful changers	45	0.6553	0.33470	0.04989	0.5548	0.7559
		Initial degree	242	0.6464	0.32970	0.02119	0.6046	0.6881
		Initial Degree + follow up	58	0.6524	0.31605	0.04150	0.5693	0.7355
		Total	530	0.6383	0.31674	0.01376	0.6113	0.6654
Lang	Social congruence	No Degree	793	0.3266	0.17245	0.00612	0.3145	0.3386
		Explicit dropout	87	0.3915	0.18144	0.01945	0.3528	0.4301
		Successful changers	297	0.3396	0.18822	0.01092	0.3181	0.3611
		Initial degree	520	0.3374	0.17175	0.00753	0.3226	0.3522
		Initial Degree + follow up	160	0.3221	0.16607	0.01313	0.2961	0.3480
		Total	1857	0.3343	0.17515	0.00406	0.3264	0.3423
	Aspirational congruence	No Degree	732	0.6562	0.28086	0.01038	0.6358	0.6766
		Explicit dropout	78	0.7223	0.34643	0.03923	0.6442	0.8004
		Successful changers	272	0.7039	0.27767	0.01684	0.6708	0.7371
		Initial degree	495	0.6549	0.24985	0.01123	0.6329	0.6770
		Initial Degree + follow up	147	0.6528	0.27457	0.02265	0.6081	0.6976
		Total	1724	0.6661	0.27514	0.00663	0.6531	0.6791

5 Supplement 5: Study satisfaction

5.1 Supplement 5a: Correlations between Social congruence (SOC) and Aspirational congruence (ASP) and satisfaction measures for all students and split for the different study areas. Lower values indicate higher congruence.

**W3**	STEM-L	STEM-M	Med	Eco	Edu	Lang
Study Satisfaction	-.004/-.023	.010/.052*	.053/-.006	-.006/-.065	-.091*/-.007	-.015/-.019
Exhausted	.031/.064*	.046/.006	.027/.079	.035/.009	.030/-.010	.038/-.003
Study Conditions	-.045*/-.092**	-.016/.048	.010/-.063	-.007/-.005	-.040/.020	-.057*/.045
**W5**						
Study Satisfaction	-.018/-.002	-.037/.049	.030/-.038	.045/-.035	-.045/-.049	-.023/-.007
Exhausted	.070*/.049	.068**/-.009	.111*/.042	.044/.105*	.063/-.039	.031/.041
Study Conditions	-.051*/-.077**	-.069**/.067*	.025/-.023	-.078*/-.059	-.007/-.014	.069**/.035

Note. Social congruence/Aspirational congruence; *: $p < .05$; **: $p < .01$

5.2 Supplement 5b: Correlations between Social congruence (SOC) and Aspirational congruence (ASP) and satisfaction measures for male students and split for the different study areas. Lower values indicate higher congruence.

**W3**	STEM-L	STEM-M	Med	Eco	Edu	Lang
Study Satisfaction	-.026/-.062*	.006/.071	.067/.042	-.069/-.172*	-.115/-.129	-.032/.024
Exhausted	.005/.062*	.023/.019	.026/.090	.064/.004	.104/.132	.078/.104
Study Conditions	-.051*/-.084**	.058/.016	.104/-.023	-.073/-.051	.104/-.139	-.097/-.018
**W5**						
Study Satisfaction	-.037/-.007	-.019/-.023	.095/-.076	.054/-.128	-.106/-.148	.065/-.025
Exhausted	.052*/.044	.068/.062	.105/.081	-.031/.165*	.129/.058	.061/.039
Study Conditions	-.058*/-.095**	.004/-.002	.020/-.095	-.078/-.111	.096/-.150	-.037/.037

Note. Social congruence/Aspirational congruence; *: $p < .05$; **: $p < .01$

5.3 Supplement 5c: Correlations between Social congruence (SOC) and Aspirational congruence (ASP) and satisfaction measures for female students and split for the different study areas. Lower values indicate higher congruence.

**W3**	STEM-L	STEM-M	Med	Eco	Edu	Lang
Study Satisfaction	.046/.111	.012/.035	.043/-.010	.032/.007	-.088/.011	-.011/-.028
Exhausted	.071/.072	.058*/.029	.045/.036	.024/.010	.022/-.028	.033/-.012
Study Conditions	.011/-.147*	-.054/.030	-.045/-.066	.030/.023	-.057/.040	-.053/.038
**W5**						
Study Satisfaction	.027/.015	-.047/.076*	.000/-.024	.040/.024	-.038/-.038	-.038/-.003
Exhausted	.096/.064	.074*/-.002	.137*/-.018	.099*/.061	.054/-.051	.028/.055
Study Conditions	-.004/-.013	-.109**/.064*	.022/.026	-.087*/-.021	-.020/.003	-.078**/.016

Note. Social congruence/Aspirational congruence; *: p < .05; **: p < .01

6 Supplement 6: Ns and 95% confidence intervals for the correlations between congruence and student's grades

6.1 Supplement 6a: Ns and 95% confidence intervals for the correlations between aspirational congruence and student's grades for the whole sample and the male and female subsamples

Group	*N*	*r*	*LCI*	*UCI*
Total	3159	.061	.026	.096
Male	1182	.071	.014	.128
Female	1977	.056	.012	.100

6.2 Supplement 6b: Ns and 95% confidence intervals for the correlations between aspirational congruence and student's grades for the different study clusters

Group	*N*	*r*	*LCI*	*UCI*
STEM-L	742	.036	-.036	.108
STEM-M	783	.127	.058	.196
MED	240	.100	-.025	.225
ECO	390	.033	-.067	.132
EDU	316	.120	.011	.229
Lang	688	.079	.004	.153

6.3 Supplement 6c: Ns and 95% confidence intervals for the correlations between aspirational congruence and student's grades for male students in the different study clusters

Group	*N*	*r*	*LCI*	*UCI*
STEM-L	595	.012	-.068	.092
STEM-M	236	.161	.037	.285
MED	78	.166	-.050	.382
ECO	159	073	-.081	.228
EDU	28	.209	-.146	.563
Lang	86	.215	.013	.416

6.4 Supplement 6d: Ns and 95% confidence intervals for the correlations between aspirational congruence and student's grades for female students in the different study clusters

Group	*N*	*r*	*LCI*	*UCI*
STEM-L	147	.136	-.023	.294
STEM-M	547	.103	.020	.186
MED	162	.071	-.082	.224
ECO	231	.003	-.126	.132
EDU	288	.114	.000	.228
Lang	602	.070	-.010	.149

6.5 Supplement 6e: Ns and 95% confidence intervals for the correlations between social congruence and student's grades for the whole sample and the male and female subsamples

Group	*N*	*r*	*LCI*	*UCI*
total	4220	.025	-.006	.055
Male	1745	.011	-.036	.058
Female	2475	.035	-.004	.074

6.6 Supplement 6f: Ns and 95% confidence intervals for the correlations between social congruence and student's grades for the different study clusters

Group	*N*	*r*	*LCI*	*UCI*
STEM-L	1246	.005	-.050	.061
STEM-M	975	.002	-.060	.065
MED	246	-.019	-.144	.106
ECO	629	.064	-.014	.142
EDU	394	.064	-.035	.162
Lang	730	.049	-.023	.122

6.7 Supplement 6h: Ns and 95% confidence intervals for the correlations between social congruence and student's grades for male students in the different study clusters

Group	*N*	*r*	*LCI*	*UCI*
STEM-L	979	.019	-.043	.082
STEM-M	320	-.010	-.119	.100
MED	80	-.176	-.388	.037
ECO	238	.032	-.095	.159
EDU	36	.211	-.101	.523
Lang	92	.033	-.171	.237

6.8 Supplement 6h: Ns and 95% confidence intervals for the correlations between social congruence and student's grades for female students in the different study clusters

Group	*N*	*r*	*LCI*	*UCI*
STEM-L	267	-.059	-.179	.061
STEM-M	655	.008	-.068	.085
MED	166	.053	-.098	.205
ECO	391	.083	-.015	.181
EDU	358	.054	-.049	.158
Lang	638	.054	-.024	.131

7 Supplement 7: Ns and 95% confidence intervals for the correlations between congruence and student's satisfaction

7.1 Supplement 7a: Ns and 95% confidence intervals for the correlations between aspirational congruence and student's satisfaction for the whole sample

Variable	*N*	*r*	*LCI*	*UCI*
Satisfaction (W3)	5630	-.008	-.034	.018
Exhaustion (W3)	5630	.041	.015	.067
Conditions (W3)	5630	.053	.027	.079
Satisfaction (W5)	5444	-.005	-.031	.022
Exhaustion (W5)	5444	.049	.023	.076
Conditions (W3)	5444	.053	.027	.080

7.2 Supplement 7b: Ns and 95% confidence intervals for the correlations between aspirational congruence and student's satisfaction for the male subsample

Aspirational congruence with Satisfaction male

Variable	*N*	*r*	*LCI*	*UCI*
Satisfaction (W3)	2021	-.043	-.086	-.001
Exhaustion (W3)	2021	.062	.019	.104
Conditions (W3)	2021	-.034	-.077	.008
Satisfaction (W5)	2058	-.040	-.083	.003
Exhaustion (W5)	2058	.078	.035	.121
Conditions (W3)	2058	-.047	-.090	-.004

7.3 Supplement 7c: Ns and 95% confidence intervals for the correlations between aspirational congruence and student’s satisfaction for the female subsample

Variable	*N*	*r*	*LCI*	*UCI*
Satisfaction (W3)	3509	.007	-.026	.040
Exhaustion (W3)	3509	.048	.015	.081
Conditions (W3)	3509	.050	.017	.083
Satisfaction (W5)	3386	.013	-.021	.046
Exhaustion (W5)	3386	.061	.027	.094
Conditions (W3)	3386	.054	.020	.087

7.4 Supplement 7d: Ns and 95% confidence intervals for the correlations between social congruence and student’s satisfaction for the whole sample

Variable	*N*	*r*	*LCI*	*UCI*
Satisfaction (W3)	7278	-.008	-.031	.014
Exhaustion (W3)	7278	.041	.018	.064
Conditions (W3)	7278	-.026	-.049	-.003
Satisfaction (W5)	7025	-.018	-.042	.005
Exhaustion (W5)	7025	.067	.043	.090
Conditions (W3)	7025	-.048	-.071	-.025

7.5 Supplement 7e: Ns and 95% confidence intervals for the correlations between social congruence and student’s satisfaction for the male subsample

Variable	*N*	*r*	*LCI*	*UCI*
Satisfaction (W3)	3012	-.025	-.061	.011
Exhaustion (W3)	3012	.022	-.013	.058
Conditions (W3)	3012	-.029	-.065	.007
Satisfaction (W5)	2912	-.015	-.051	.021
Exhaustion (W5)	2912	.048	.012	.084
Conditions (W3)	2912	-.042	-.078	-.005

7.6 Supplement 7f: Ns and 95% confidence intervals for the correlations between social congruence and student’s satisfaction for the female subsample

Variable	*N*	*r*	*LCI*	*UCI*
Satisfaction (W3)	4266	.003	-.027	.033
Exhaustion (W3)	4266	.054	.024	.084
Conditions (W3)	4266	-.024	-.054	.006
Satisfaction (W5)	4113	-.021	-.051	.010
Exhaustion (W5)	4113	.080	.050	.111
Conditions (W3)	4113	-.056	-.086	-.025
